# Extrachromosomal circular DNA orchestrates genome heterogeneity in urothelial bladder carcinoma

**DOI:** 10.7150/thno.99563

**Published:** 2024-08-19

**Authors:** Wei Lv, Xiaoguang Pan, Peng Han, Shuang Wu, Yuchen Zeng, Qingqing Wang, Lidong Guo, Mengyang Xu, Yanwei Qi, Li Deng, Zhe Xu, Conghui Li, Tianxi Yu, Xin Cui, Huajing Teng, Chongjun Xiang, Haotian Tan, Yue Li, Ning Liang, Huiying Tao, Qingqing Gao, Guohua Yu, Jia Mi, Fuyi Xu, Benjiao Gong, Lei Shi, Tao Wang, Huanming Yang, Wei Dong, Lars Bolund, Lin Lin, Wenting Wang, Hanbo Li, Jinrong Huang, Chunhua Lin, Yonglun Luo

**Affiliations:** 1 Lars Bolund Institute of Regenerative Medicine, HIM-BGI Omics Center, Hangzhou Institute of Medicine (HIM), Chinese Academy of Sciences, Hangzhou, Zhejiang 310022, China.; 2 College of Life Sciences, University of Chinese Academy of Science, Beijing 100049, China.; 3 Lars Bolund Institute of Regenerative Medicine, Qingdao-Europe Advanced Institute for Life Sciences, BGI-Research, Qingdao 266555, China.; 4 BGI-Research, Shenzhen, 518083, China.; 5 Department of Urology, The Affiliated Yantai Yuhuangding Hospital of Qingdao University, Yantai, Shandong, 264000, China.; 6 School of Clinical Medicine, Weifang Medical University, Weifang, 261042, China.; 7 Key Laboratory of Carcinogenesis and Translational Research (Ministry of Education/Beijing), Department of Radiation Oncology, Peking University Cancer Hospital & Institute, Beijing, China.; 8 The 2nd Medical College of Binzhou Medical University, Yantai, Shandong, 264003, China.; 9 Department of Urology, China-Japan Friendship Hospital (Institute of Clinical Medical Sciences), Chinese Academy of Medical Sciences & Peking Union Medical College, Beijing, 100029, China.; 10 Department of Pathology, The Affiliated Yantai Yuhuangding Hospital of Qingdao University, Yantai, Shandong, 264000, China.; 11 Shandong Technology Innovation Center of Molecular Targeting and Intelligent Diagnosis and Treatment, School of Pharmacy, Binzhou Medical University, Yantai, Shandong, China.; 12 Department of Central Laboratory, The Affiliated Yantai Yuhuangding Hospital of Qingdao University, Yantai, Shandong 264000, China.

**Keywords:** urothelial bladder carcinoma, extrachromosomal circular DNA, eccDNA, ecDNA, cancer genetics

## Abstract

**Rationale:** Extrachromosomal circular DNA is a hallmark of cancer, but its role in shaping the genome heterogeneity of urothelial bladder carcinoma (UBC) remains poorly understood. Here, we comprehensively analyzed the features of extrachromosomal circular DNA in 80 UBC patients.

**Methods:** We performed whole-genome/exome sequencing (WGS/WES), Circle-Seq, single-molecule real-time (SMRT) long-read sequencing of circular DNA, and RNA sequencing (RNA-Seq) on 80 pairs of tumor and AT samples. We used our newly developed circular DNA analysis software, Circle-Map^++^ to detect small extrachromosomal circular DNA from Circle-Seq data.

**Results:** We observed a high load and significant heterogeneity of extrachromosomal circular DNAs in UBC, including numerous single-locus and complex chimeric circular DNAs originating from different chromosomes. This includes highly chimeric circular DNAs carrying seven oncogenes and circles from nine chromosomes. We also found that large tumor-specific extrachromosomal circular DNAs could influence genome-wide gene expression, and are detectable in time-matched urinary sediments. Additionally, we found that the extrachromosomal circular DNA correlates with hypermutation, copy number variation, oncogene amplification, and clinical outcome.

**Conclusions:** Overall, our study provides a comprehensive extrachromosomal circular DNA map of UBC, along with valuable data resources and bioinformatics tools for future cancer and extrachromosomal circular DNA research.

## Introduction

Urothelial bladder carcinoma (UBC) is a common and highly heterogeneous urologic malignancy, accounting for over 90% of bladder cancer cases. According to the depth of tumor invasion, UBC is classified into muscle-invasive bladder cancer (MIBC; pT2-pT4) and superficial non-MIBC (NMIBC; pTa/1), each with distinct molecular and clinical characteristics 1-3. UBC is also known for its extremely high somatic mutation burden 4, 5. Despite advancements in surgical methods and treatments, such as anti-PD1 and PD-L1 immunotherapy, patient outcomes remain suboptimal, and the incidence rate has been rising in recent years 6-8. Therefore, a comprehensive understanding of the genetic and molecular factors driving the development, progression, and diversification of genomic mutations and alterations in UBC is essential.

Extrachromosomal circular DNA was initially identified in boar sperm and wheat embryos in 1964 by Yasuo Hotta and Alix Bassel 9. These chromosome-originated, centromere-free, and plasmid-like DNA species have recently gained increasing attention due to their important roles in carcinogenesis 10-17. Extrachromosomal circular DNA is now commonly categorized into two main types: 1) large, cancer-specific extrachromosomal circular DNA with copy number amplification, often referred to as ecDNA 13, and 2) small, non-mitochondrial extrachromosomal circular DNA (eccDNA, including microDNA)^18^, ^19^. These two types of DNA, ecDNA and eccDNA, exhibit significant structural and functional differences, which is why they are usually discussed separately in research. ecDNA, first discovered in childhood cancer cells in 1965 20, 21, is found in a subset of tumors across various cancer types and is closely linked to increased oncogene dosage 22. The widely accepted model for ecDNA-based amplification is asymmetric segregation 23. During cell mitosis, ecDNA is replicated but distributed unevenly to the two daughter cells due to the absence of centromeres 23. This inheritance model leads to the rapid accumulation of ecDNA in cancer cells. Moreover, ecDNA can achieve transcriptional heterogeneity by forming ecDNA hubs 23, 24. Thus, targeting ecDNA molecules may offer an appealing therapeutic strategy for cancer patients with ecDNA-containing tumors 25. Kim et al. 22 revealed the characteristics and distribution of ecDNA in the pan-cancer study but did not particularly emphasize its role in UBC. Moreover, the presence of ecDNA in urine samples of UBC is largely unknown.

Extrachromosomal circular DNAs with smaller sizes (eccDNAs) are widely detected in normal, benign, and tumor tissues 15, 26-28. EccDNAs appear to arise from all parts of the genome and can carry various genetic elements, ranging from small, non-coding gene fragments to entire protein-coding gene sequences 15, 19, 27. They are particularly enriched in GC-rich and repetitive genomic regions and have been associated with diverse cellular functions, including activation of endogenous immune responses 29, 30, serving as templates for the transcription of small regulatory RNA 31, aiding in environmental adaption 32, and contributing to genome stability 26. Therefore, it is reasonable to hypothesize that eccDNAs affect cancer phenotype. However, very little is known about its characteristics in cancers such as UBC, and their roles in contributing to genomic heterogeneity. Here, using a multi-omics strategy and our newly developed circular DNA analysis software, Circle-Map^++^, we systematically map the extrachromosomal circular DNA (ecDNA/eccDNA) landscape in UBC and assess its potential influence on genome remodeling, gene expression, and patient outcomes. Our integrative extrachromosomal circular DNA profiling not only adds new insights into our understanding of the role of circular DNA in bladder cancer but also provides valuable data resources and bioinformatics tools for future cancer and circular DNA research.

## Results

### Study samples

The CCGA-UBC project enrolled 80 UBC patients, each providing matched freshly snap-frozen tumor tissues, and tumor-adjacent tissues (ATs) (**Table 1 and Figure S1A**). Of these patients, approximately 73% (58/80) had MIBC, which is considerably higher than that in the general UBC population (20%) 7. Patients with MIBC showed poorer clinical outcomes compared to those with NMIBC, though statistical significance was not reached due to the limited sample size (log-rank test; Overall survival (OS), *P* = 0.1696; progression-free survival (PFS), *P* = 0.1196) (**Figure S1B**). Among the patients, 84% (67/80) were male and the median age at diagnosis was 68 years (range: 47-87). Furthermore, 85% of patients were diagnosed with high-grade UBC, and 88% underwent radical cystectomy (**Figure S1C**). Detailed clinical and pathological characteristics of the included CCGA-UBC patients are provided in **Table S1**.

**Figure 1:**
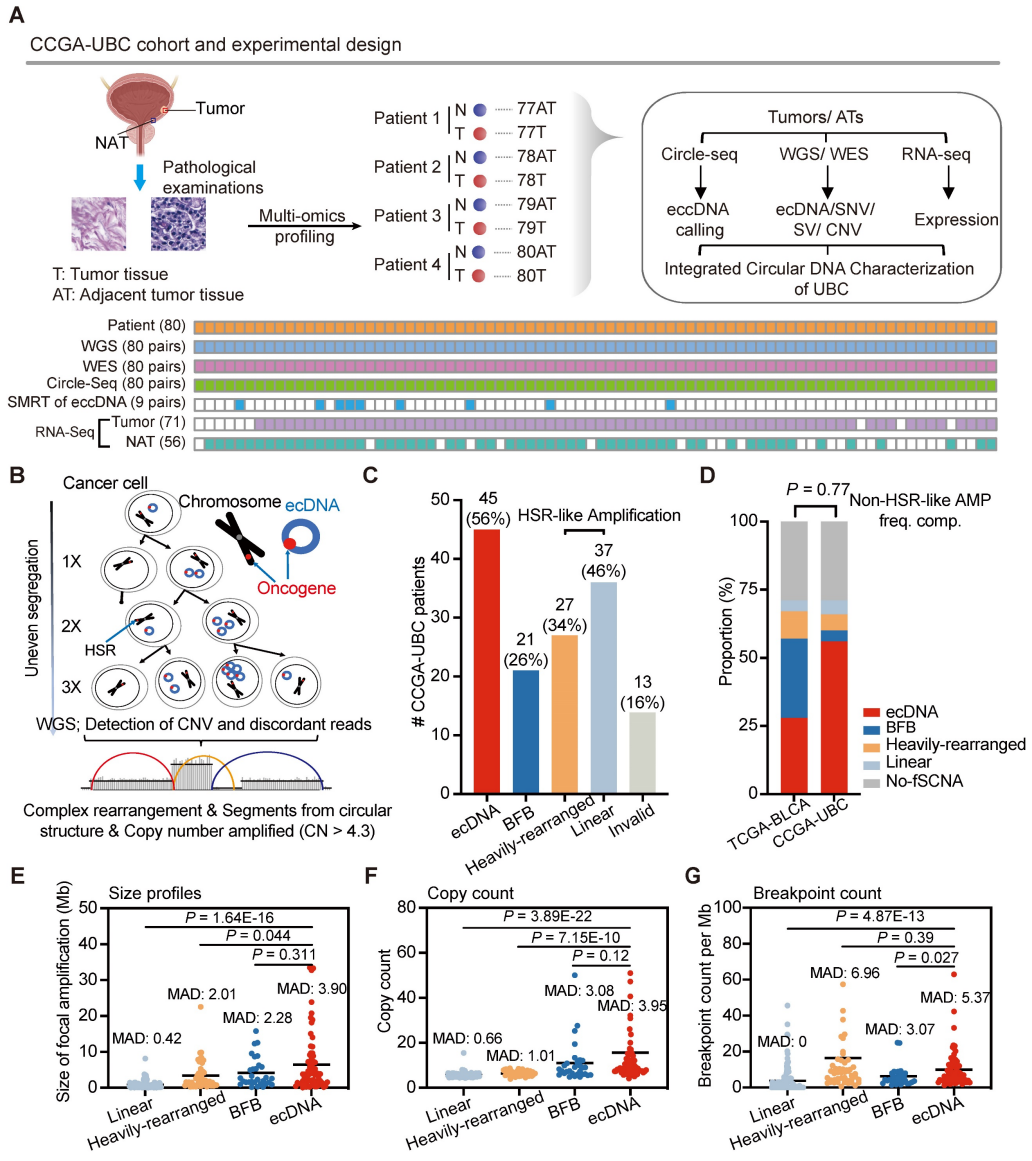
** Experimental workflow and comprehensive ecDNAprofiling in CCGA-UBC samples (n = 80 patients)**. (A) Experimental workflow. A total of 80 pairs of freshly snap-frozen tumor tissues and Adjacent Tumor Tissues (ATs) were collected from UBC patients. Each sample underwent WGS, WES, Circle-Seq, SMRT of eccDNA, and RNA-seq for comprehensive extrachromosomal circular DNA analysis. WGS, WES, and Circle-Seq, were performed for all 80 pairs of tumor-AT samples. SMRT of eccDNA was performed on 9 pairs of tumor-AT samples. RNA samples from 9 tumor tissues and 24 ATs were excluded due to low RNA quality. (B) Model illustrating rapid accumulation of ecDNAs in cancer. Unlike chromosomes, ecDNA lacks centromeres, meaning their separation during mitosis is not controlled by the mitotic spindle. Consequently, ecDNAs are randomly distributed into daughter cells during cell division. This non-Mendelian inheritance pattern causes intra-tumoral heterogeneity. ecDNAs were detected using AA software based on copy number data and discordant reads inferred from WGS data. Abbreviations: HSR, homogenously staining region. (C) Number of UBC patients with indicated amplicon types in the CCGA-UBC cohort. Amplicons were classified as ecDNA, BFB, Heavily-rearranged, and Linear. Among these, Linear and Heavily-rearranged types are classified as HSR-like amplification. (D) Frequency of each focal amplification type between the TCGA-BLCA cohort and CCGA-UBC cohort. The frequency of non-HSR-like amplification (ecDNA and BFB) was compared using Fisher's exact test. (E-G) Comparison of size (E), copy count (F), and breakpoint count (G) for each type of focal amplification (Linear, n = 124; Heavily-rearranged, n = 49; BFB, n = 30; ecDNA, n = 68; Wilcoxon rank-sum test). The median absolute deviation (MAD) score is indicated.

To comprehensively characterize the extrachromosomal circular DNA landscape in UBC and gain insights into its role in genomic heterogeneity and instability, we performed whole-genome/exome sequencing (WGS/WES), Circle-Seq, single-molecule real-time (SMRT) long-read sequencing of circular DNA, and RNA sequencing (RNA-Seq) on 80 pairs of tumor and AT samples (**Figure 1A and Table S2**). Consistent with previous reports 4, 5, we observed a high tumor mutation load of UBC samples (**Figure S1D**).

### ecDNA is common in UBC and exhibits distinct genomic patterns with homogenously staining region (HSR)-like amplifications

Unbalanced structural variations resulting from DNA circularization play a significant role in carcinogenesis 10, 11, 15, 22. However, their genome-wide frequency, structure, composition, and heterogeneity in UBC have not been comprehensively assessed. Currently, it is generally recognized that extrachromosomal circular DNA falls into two primary types: 1) large, cancer-associated extrachromosomal circular DNA exhibiting copy number amplification, commonly denoted as ecDNA 13, and 2) small, non-mitochondrial extrachromosomal circular DNA, abbreviated as eccDNA. 18, 19 (**Figure S2A**).

We first explored the prevalence of ecDNA in UBC. Using the WGS data generated in this study, we applied the AmpliconArchitect (AA) 33 software to quantify and characterize the focal amplifications in UBC (**Figure 1B**). The AA tool is known for its ability to predict ecDNA with 83% sensitivity and 85% precision from WGS data 22. Focal amplifications can be categorized into ecDNA, Breakage-Fusion-Bridge (BFB), Linear amplification, and Heavily-rearranged. Although ecDNA and BFB are linked to well-established mechanisms, the other two types resemble homogeneously staining region (HSR) amplifications and lack clearly defined formation mechanisms 33. Of note, the current computational methodology faces significant difficulties in identifying BFB and ecDNA. We found that focal amplifications were present at high frequencies (71%, 57/80) within the CCGA-UBC cohort. Specifically, ecDNA was identified in 56% (45/80) of UBC tumor samples, indicating its common occurrence in UBC (**Figure 1C; Table S3**). In 62.2% of ecDNA-positive (ecDNA^+^) cases, there was only one distinct ecDNA, with just four patients carrying more than two ecDNA species (**Figure S2B**). Moreover, 40 patients displayed multiple types of focal amplifications (**Table S4**). The frequency of non-HSR-like amplifications (ecDNA and BFB) in our cohort (60%) is similar to that previously reported by Kim *et al*. in the TCGA-bladder cancer cohort (57%) (Fisher's exact test, *P* = 0.77) (**Figure 1D**).

Each type of focal amplification displayed a broad distribution across the genome (**Figure S2C**). To gain a deeper understanding of the genomic characteristics of ecDNA, we compared its size, copy number, and breakpoint count to those of HSR-like amplifications. ecDNA spanned a wide range of genomic scales and achieved a higher copy number than HSR-like amplifications (Wilcoxon rank-sum test; size profiles: *P* = 1.64E-16 for Linear and *P* = 0.044 for Heavily-rearranged; Copy count: *P* = 3.89E-22 for Linear and *P* = 7.15E-10 for Heavily-rearranged) (**Figures 1E and 1F**). We also observed that ecDNA had a higher number of breakpoints than Linear amplification (Wilcoxon rank-sum test;* P* = 4.87E-13) (**Figure 1G**). Furthermore, ecDNA demonstrated greater variability in both size and copy number, as indicated by the Median Absolute Deviation (MAD) score, compared to HSR-like amplifications. These data suggest that ecDNA exhibits distinct genomic patterns with HSR-like amplifications.

### Diversity of oncogenes encoded on ecDNA

Unlike chromosomal DNA, ecDNA elements lack centromeres, resulting in uneven segregation from parental cancer cells to offspring cells during mitosis 23. This non-chromosomal inheritance mechanism facilitates the rapid accumulation of growth-promoting ecDNAs with oncogenes in cancer cells, contributing to intra-tumoral heterogeneity.

Therefore, we focused our analysis on ecDNAs carrying oncogenes. We found that 39.1% (36/92) of ecDNAs carried oncogenes, and oncogene-bearing ecDNA was present in 66.67% (30/45) of ecDNA^+^ UBC cases (**Tables S5, 6**). A series of well-known oncogenes, including *PABPC1, CCND1, UBR5, FBXO43*, and *MDM2,* were found amplified on ecDNA in both CCGA-UBC and TCGA-BLCA cohorts (**Figure S2D**). Notably, the co-selection of oncogenes within ecDNAs was observed. For instance, tumor sample CCGA-UBC-034T contained an ecDNA with seven genes, including *CDK4, DDIT3, GLI1, HMGA2, PTPN11, RFC5,* and* TMPO* (**Table S5**). Some of these oncogenes found in ecDNAs are clinically relevant; for example, an ecDNA in CCGA-UBC-015T contained the *FGFR3* gene (**Table S5**). The *FGFR* tyrosine kinase inhibitor (TKI) is currently an attractive treatment for advanced UBC patients. Meanwhile, we observed that the ecDNA structures were largely sample-specific and could originate from multiple chromosomes. For example, tumor sample CCGA-UBC-001T contained an ecDNA with just one segment, while tumor sample CCGA-UBC-084T carried a complex multi-chromosomal ecDNA (**Figures S2E, F**). Metaphase DNA fluorescence in situ hybridization (FISH) is currently considered the gold-standard method for distinguishing oncogenes amplified on HSR or ecDNA. However, it is only applicable to cell line samples. Therefore, we performed interphase FISH on four available tumor samples with *CCND1*-ecDNA (**Figure S3A**). We observed abundant scattered amplification signals within the nuclei (**Figure S3B**). Although interphase FISH is not the gold standard, these results still reflect, to some extent, the characteristic high copy number of genes carried by ecDNA. Overall, our analysis highlights the diversity of ecDNA structures and their harboring of oncogenes.

### ecDNA is common in urine sediment samples of patients with ecDNA^+^ Tumors

**Figure 2:**
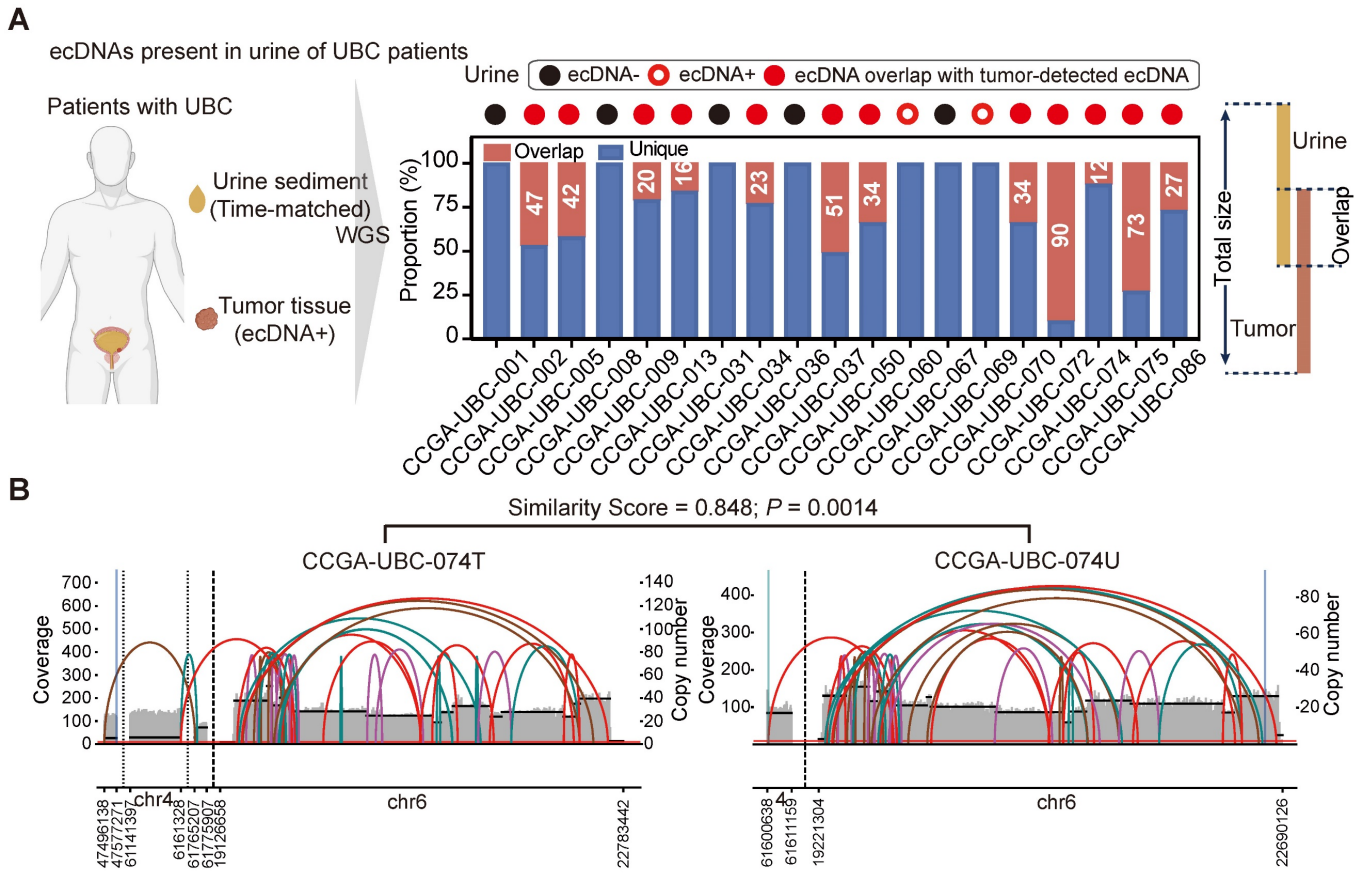
** ecDNA is common in urine sediment samples of patients with ecDNA+ tumors (n = 19 patients).** (A) ecDNAs present in the urine sediment samples. The left panel illustrates the workflow for detecting ecDNA in urine sediment samples. WGS was performed on time-matched urine samples from 19 patients with ecDNA^+^ tumors. The right panel shows the overlap of ecDNA detected in tumors and the corresponding urine samples. (B) Analysis of AA-generated structural variant (SV) and breakpoint graphs: Examples of ecDNA detected in tumors and corresponding time-matched urine samples from patient CCGA-UBC-074 are shown. The *P* value was calculated based on similarity scores among genomic overlapped ecDNA from tumors and matched urine samples using a beta-distribution model.

We next aimed to determine whether tumor-specific ecDNA could be identified in urine sediment samples of UBC patients. We performed low-depth WGS on time-matched urine sediment samples from 19 UBC patients with ecDNA^+^ tumors (**Figure 2A, left panel; Table S2**). EcDNA was detected in 14 of the 19 (74%) urine sediments analyzed.

Twelve urine samples exhibited ecDNA that overlapped with the ecDNA regions identified in their corresponding tumors (**Figure 2A, right panel**), and some ecDNAs found in urine samples displayed similar structures to those in their matched tumors (**Figure 2B; Table S7**). Notably, we found that the ecDNA detected in the urine of two patients (CCGA-UBC-060/069) was entirely different from the ecDNA identified in their tumor tissues (**Figure 2A, right panel**). This discrepancy may be attributed to the intratumoral heterogeneity of ecDNA. Our results demonstrate for the first time that tumor-specific ecDNA is common in urine samples of patients with ecDNA^+^ tumors, and urinary ecDNAs share some common features of tumor-derived ecDNA.

### Heterogeneity of small extrachromosomal circular DNA (eccDNA) in UBC

Small extrachromosomal circular DNAs (including microDNAs, eccDNAs), are more commonly identified in tissues compared to larger amplified-ecDNAs 17, 19. However, the characteristics of eccDNA in most cancers such as UBC remain poorly understood. To gain more insights into eccDNA heterogeneity in UBC, we used Circle-Seq to analyze the 80 paired UBC tumors and ATs (**Figure S4A**). Circle-Seq 18 is a sequencing library enrichment approach that enables genome-wide detection of eccDNAs. To enhance data processing and eccDNA mapping speed, we developed a new bioinformatics pipeline, Circle-Map^++^, which detects eccDNAs from Circle-Seq data using soft-clipped reads and discordant read pairs. Compared with Circle-Map 34, Circle-Map^++^ identifies more eccDNAs and offers advantages in time efficiency and detection accuracy (**Figure S4B**).

**Figure 3:**
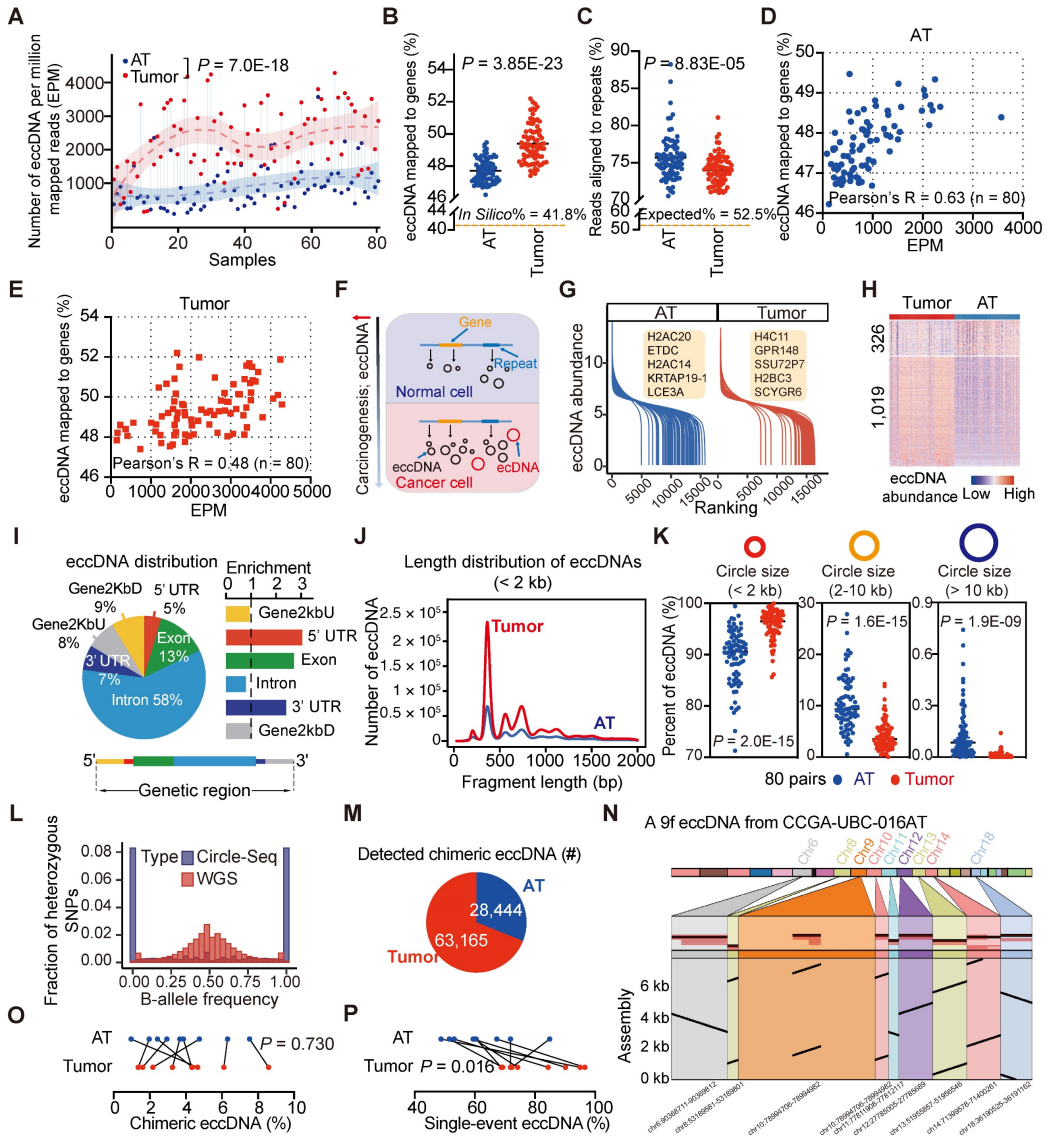
** Differential patterns of small extrachromosomal circular DNA (eccDNA) between tumors and ATs (n = 80 patients).** (A) Comparison of eccDNA counts per million mapped reads (EPM) between CCGA-UBC tumors and ATs (80 pairs; Paired t-test). eccDNAs were identified using a combination of Circle-Seq and Circle-Map^++^ methods. (B) Percentage of eccDNA mapped to protein-coding genes relative to all detected eccDNAs in tumors and ATs (80 pairs; Paired t-test). (C) Percentage of repeats in eccDNA-enriched datasets from tumors and ATs (80 pairs; Paired t-test). (D-E) Scatter plots showing the relationship between the EPM and the percentage of eccDNA mapped to protein-coding genes in ATs (D) and Tumors (E) (Pearson correlation test, n = 80). (F) A brief model depicting changes in the eccDNA profile during bladder carcinogenesis. (G) Overview of the eccDNA profile in CCGA-UBC samples. The graph illustrates the dynamics of eccDNA abundance of protein-coding genes in ATs (blue) and tumors (red). The eccDNA abundance for each protein-coding gene was calculated based on the unique junction (start point) counts, normalized by gene length and the number of detected eccDNAs. (H) Heatmap showing differential eccDNA abundance levels in protein-coding genes between tumors and ATs (80 pairs; absolute log_2_ fold change > 0.5; Wilcoxon rank-sum test, *P* < 0.01). (I) Genomic annotation of tumor-derived eccDNAs. The left panel shows the fraction of genomic elements affected by eccDNA, while the right panel shows the relative enrichment of eccDNA in each genomic element. The bottom panel briefly illustrates the location of genomic elements on the gene body. (J) Length distribution of eccDNAs (< 2 kb) detected in tumors and ATs (Pooled data from all cases in each group). (K) Comparison of the percentage of eccDNA across length ranges (< 2 kb; 2-10 kb; > 10 kb) in tumors and ATs (80 pairs; Paired t-test). (L) Distribution of alternative-B-allele frequency (BAF) in sequencing reads from WGS and Circle-Seq (n = 80 tumors). Most eccDNAs are of mono-allelic origin. (M) Pie chart showing the number of chimeric eccDNA in tumors and ATs. Chimeric eccDNAs refer to eccDNAs consisting of multiple fragments from one or more chromosomes. (N) Assembled sequence of a nine-fragment eccDNA (9f eccDNA) in CCGA-UBC-016N. (O) Comparison of the percentage of chimeric eccDNA among the total unique eccDNAs in tumors and ATs (9 pairs; Paired t-test). (P) Comparison of the percentage of single-event eccDNAs in tumors and ATs (9 pairs; Paired t-test). Single-event eccDNA refers to eccDNA that was sequenced from only a single long-read.

Our Circle-Seq results revealed significant heterogeneity in eccDNA levels among UBC samples. Tumor samples contained significantly higher levels of eccDNA compared to AT samples, with a median of 540,972 (range: 34,595-1,741,398) eccDNAs detected per UBC tumor sample and 131,764 (range: 20,052-1,313,643) eccDNAs per AT sample (**Table S8**). To account for sequencing depth, we applied a normalized eccDNA quantification by calculating the eccDNA count per million mapped reads (EPM). The EPM value was 2.6-fold higher in UBC tumor tissues than in ATs (Paired t-test, *P* = 7.0E-18) (**Figure 3A**), consistent with the previous findings suggesting that carcinogens can elevate eccDNA levels 35. Interestingly, tumor-derived eccDNAs were found to accumulate in protein-coding genes more frequently than those from ATs (*In Silico*: 41.8%; Tumors, median: 49.2%, range: 47.4%- 52.2%; ATs, median: 47.6%, range: 46.2%- 49.5%) (Paired t-test, *P* = 3.85E-23) (**Figure 3B; Table S8**). We also extracted eccDNA sequences from repetitive DNA of the human genome. The percentage of tumor-derived eccDNA reads (median: 74.2%; range: 69.0%-81.1%) that aligned to repetitive regions was significantly lower compared to AT-derived eccDNA reads (median: 75.1%; range: 70.5%-88.2%) (Paired t-test, *P* = 8.83E-05) (**Figure 3C; Table S8**). Furthermore, we found a positive correlation between the eccDNA level and the percent of eccDNA mapped to protein-coding genes (AT, Pearson's R = 0.63, *P* = 5.66E-10; Tumor, Pearson's R = 0.48, *P* = 5.69E-06) (**Figures 3D and 3E**). These findings suggest that eccDNA levels increase during tumor progression, with elevated eccDNA more likely to be enriched in protein-coding regions (**Figure 3F**).

We then performed differential eccDNA profile analysis on paired tumors and ATs. Given the high correlation between the number of start and end sites of eccDNA in each gene (e.g., CCGA-UBC-001T; Person's R = 0.97, *P* < 2.2E-16) (**Figure S4D**), we quantified eccDNA abundance as the number of unique start sites in a specific gene normalized by the gene length and the total number of mapped eccDNAs. The eccDNA abundance in tumors and ATs did not follow a normal distribution, and an average of 21% (range: 7%-72%) of genes per sample belonged to an “eccDNA desert” region, with no eccDNA instances (**Figure S4E**). The dynamics of eccDNA abundances identified in ATs and tumors are shown in **Figure 3G**. Genome-wide comparison of eccDNA profiles between tumors and ATs revealed 1,345 genes with significantly different levels of eccDNA abundance. Among these, 1,019 genes exhibited higher eccDNA abundance in tumors compared to ATs (absolute log_2_ fold change > 0.5; Wilcoxon rank-sum test, *P* < 0.01) (**Figure 3H**). Notably, 74 of these genes overlapped with well-known cancer-associated genes (CAGs), such as *CCR4*, *KLF4*, *FANCG*, and *NAB2* (**Table S9**). For instance, *CCR4* showed significantly higher eccDNA levels in tumors compared to ATs (Wilcoxon rank-sum test, *P* = 7.24E-07, **Figure S4F**). Three typical examples of *CCR4* eccDNAs were 3,524 bp, 3,528 bp, and 2,667 bp in length, covering more than 50% of the *CCR4* gene body (**Figure S4G**). Further validation using out-ward PCR and Sanger sequencing confirmed the presence of these three *CCR4* eccDNAs in tumor samples (**Figure S4H and Table S10**). Moreover, gene ontology (GO) analysis of these 1,345 genes with differential eccDNA abundance showed the enrichment in GO terms related to cancer- and immune-related signatures, such as inflammatory response, IL-17 signaling pathway, and TNF signaling pathway (**Figure S4I**), suggesting a potential role of eccDNA in the pathogenesis of UBC.

We next investigated the genomic and sequence features of eccDNAs. The eccDNA distribution was not restricted to specific genomic regions (**Figure 3F**). Predominantly, eccDNAs were observed in the intronic region and GC-rich environment (**Figures 3I and S4C; Table S8**). However, when normalized to genomic element lengths, the greatest enrichment of eccDNAs was found in the untranslated regions (UTRs) and coding sequences (**Figure 3I**). The size distribution of eccDNAs displayed a “ladder” pattern and varied significantly between tumor tissues and ATs (**Figure 3J**). Specifically, tumor tissues tended to contain a higher proportion of small eccDNAs compared to ATs [Paired t-test; circle size < 2 kb, tumors (median: 96.5%; range: 85.62%-100%), ATs (median: 90.6%, range: 71.25%-98.49%), *P* = 2.0E-15; circle size 2-10 kb, tumors (median: 3.49%, range: 0.03%-14.24%), ATs (median: 9.35%, range: 1.51%-27.80%), *P* = 1.6E-15); circle size > 10 kb, tumors (median: 0.01%, range: 0%-0.14%), ATs (median: 0.16%, range: 0%-0.95%), *P* = 1.9E-09] (**Figure 3K**). Furthermore, whole-genome haplotype analysis revealed that eccDNAs detected in UBC were mainly of mono-allelic origin (**Figure 3L**). These fundamental characteristics of eccDNAs align closely with previous findings in somatic tissues 27 and neuroblastoma 17, suggesting a high degree of conservation across different tissue types.

EccDNAs may comprise multiple genomic fragments from a single or different chromosome, termed chimeric eccDNAs 26. To further investigate the chimeric eccDNA status in UBC, we sequenced 9 pairs of UBC tumors and ATs with Single-molecule real-time (SMRT) long-read sequencing (PacBio). We generated a median of 443,330 long reads per sample with a mean size range from 3.7 kb to 7.6 kb (**Table S11**). To enhance confidence in eccDNA calling, we selected long-reads that passed ≥ 2 times coverage of the entire circularized region for further analysis (**Table S12**). While most of these non-chimeric eccDNAs were also detected by short-read deep sequencing, the lower sequencing depth of SMRT only captured a small number of circles (**Figure S4J; Table S13**). The mean size of eccDNAs detected by SMRT (median: 1239.2 bp, range: 909.3 bp-1716.4 bp) was significantly longer than that detected by Circle-Seq (median: 847.1 bp, range: 580.7 bp-1203.7 bp), highlighting complementarity between these two techniques (Paired t-test, *P* = 3.92E-07, **Figure S4K**). Meanwhile, we observed a total of 63,165 and 28,444 chimeric eccDNAs in tumors and ATs respectively (**Figure 3M**). These chimeric eccDNAs are randomly ligated between chromosomes and are not limited to the same chromosome (**Figure S5L**). In one extreme case, we identified a 5.1 kb eccDNA in CCGA-UBC-016N carrying DNA fragments originating from nine chromosomes (**Figure 3N**). The percentage of chimeric eccDNAs between tumors and ATs did not exhibit a significant difference (Paired t-test, *P* =0.730) (**Figure 3O**). We noticed that most eccDNAs were obtained from a single unique long molecule (single-event eccDNA), indicating a high degree of randomization in eccDNA generation (**Figure S4M**). Tumors showed a higher percentage of single-event eccDNAs than ATs (Paired t-test, *P* =0.016) (**Figure 3P**), further indicating the increased genomic heterogeneity and instability in the UBC cancer genome.

### Correlation between ecDNA/eccDNA and gene expression

**Figure 4:**
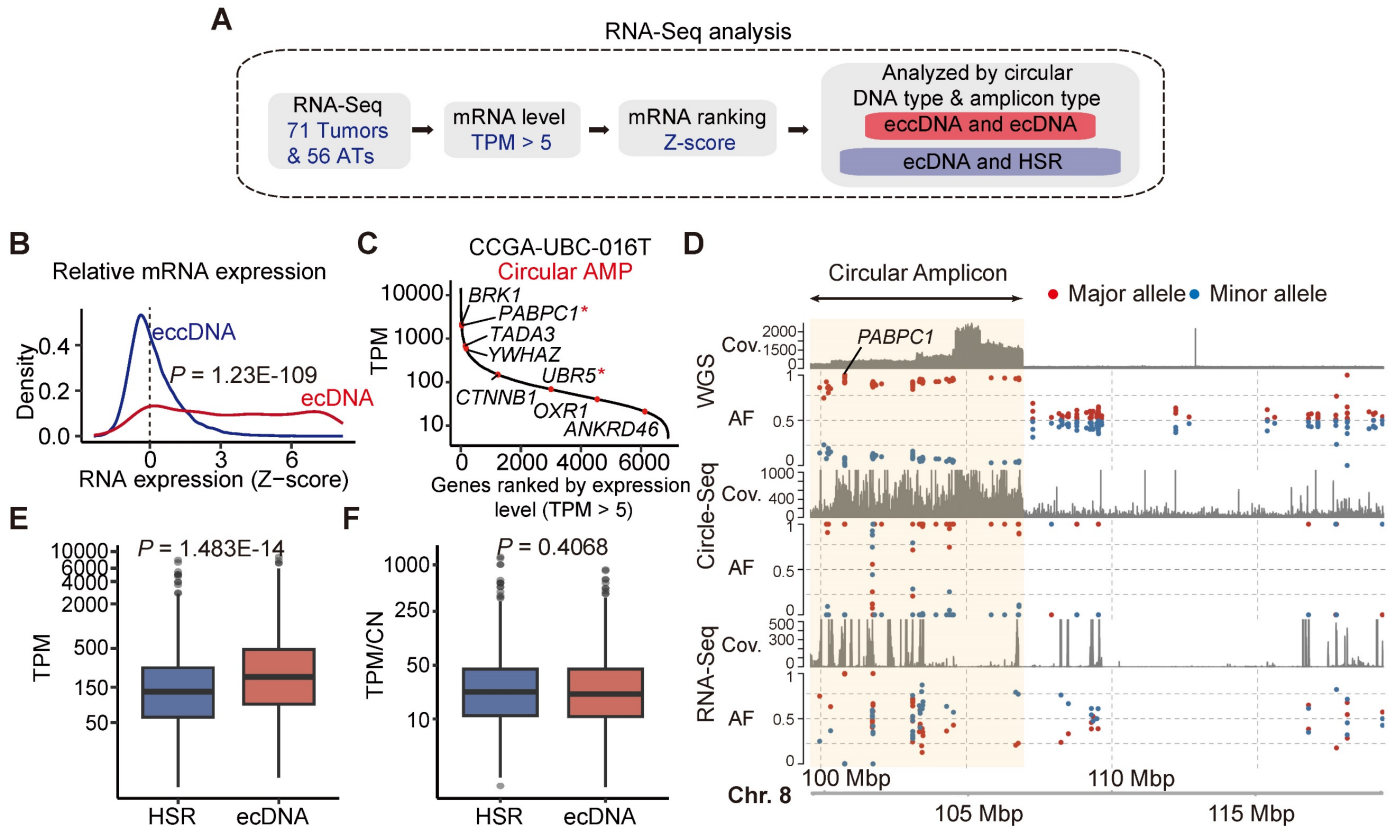
** Correlation between ecDNA/eccDNA and gene expression.** (A) Workflow for mRNA expression analysis. (B) Relative mRNA expression (Z-scores) of genes encoded on eccDNA and ecDNA (Wilcoxon rank-sum test). (C) Ranked mRNA expression in the CCGA-UBC-016 tumor sample. Red dot indicates genes carried on ecDNA. Oncogenes are marked with an asterisk (“*”). (D) Allele-specific analysis and genome browser tract at the *PABPC1* gene locus from WGS, RNA-Seq, and Circle-Seq. The circular amplicon region is highlighted in yellow. Abbreviations: AF, allele frequency. (E-F). Comparison of gene expression levels (Transcripts Per Million; TPM) (E) and gene expression levels normalized by copy number (TPM/CN) (F) between genes encoded on ecDNA and HSR-like amplification (Heavily-rearranged and Linear) (Wilcoxon rank-sum test).

We next investigated the correlation between extrachromosomal circular DNAs and gene expression (**Figure 4A**). Our analysis revealed that the expression of most genes detected in ecDNA was increased (**Figure 4B**). For instance, *PABPC1*, the most frequent ecDNA-harbored gene detected in our CCGA-UBC cohort, was highly expressed in sample CCGA-UBC-016T (**Figure 4C**). To further validate this, we performed an allele-specific expression (ASE) analysis of *PABPC1* and confirmed that the increased mRNA abundance originated from the amplified-circular allele (**Figure 4D**). In contrast, most genes associated with eccDNA fragments showed minimal changes in their expression (Wilcoxon rank-sum test; ecDNA versus eccDNA, *P* =1.23E-109) (**Figure 4B**). Only a few genes with eccDNA were detected overexpressed. One such example is the ferritin light polypeptide-1 (*FTL*) gene, which exhibited high eccDNA and mRNA abundance in sample CCGA-UBC-065T, with no significant alteration observed in the copy number of *FTL* (**Figure S5A**).

We then explored the transcriptional differences between ecDNA and HSR-like amplification (linear and Heavily-rearranged). Our data revealed that genes encoded on ecDNA produced more transcripts than those on HSR-like amplification (Wilcoxon rank-sum test, *P* = 1.483e-14) (**Figure 4E**). To evaluate gene activity, we normalized the expression level by gene copy numbers. Our results showed that genes on ecDNA exhibited similar mRNA expression levels to those on HSR-like amplification. (**Figure 4F**; Wilcoxon rank-sum test, *P* = 0.4068) These data suggested that the increased mRNA level is mostly attributable to the increased copy number dosage of ecDNA.

To investigate the relationship between eccDNA abundance and mRNA expression at the gene level, we calculated the correlation between 6,918 mRNA-eccDNA pairs in AT/tumor samples using Spearman's correlation coefficient. Both tumors and ATs exhibited relatively low mRNA-eccDNA correlation values, with a median of 0.150 in tumors and a lower median of 0.031 in ATs (**Figure S5B; Tables S14, 15**). In tumors, 1,930 mRNA-eccDNA pairs showed a significant positive correlation, predominantly associated with nucleocytoplasmic transport and ribonucleoprotein complex biogenesis (**Figure S5C**). In ATs, 284 mRNA-eccDNA pairs were significantly positively correlated with pathways involved in protein folding and DNA double-strand break processing (**Figure S5C**). Additionally, we identified 301 differentially expressed genes (DEGs) with abnormal eccDNA abundance in UBC (**Figure S5D**). Our data indicates that, at the bulk level, there is no clear correlation between eccDNA abundance and mRNA expression.

### Frequent co-occurrence of ecDNA with other genomic alterations

**Figure 5:**
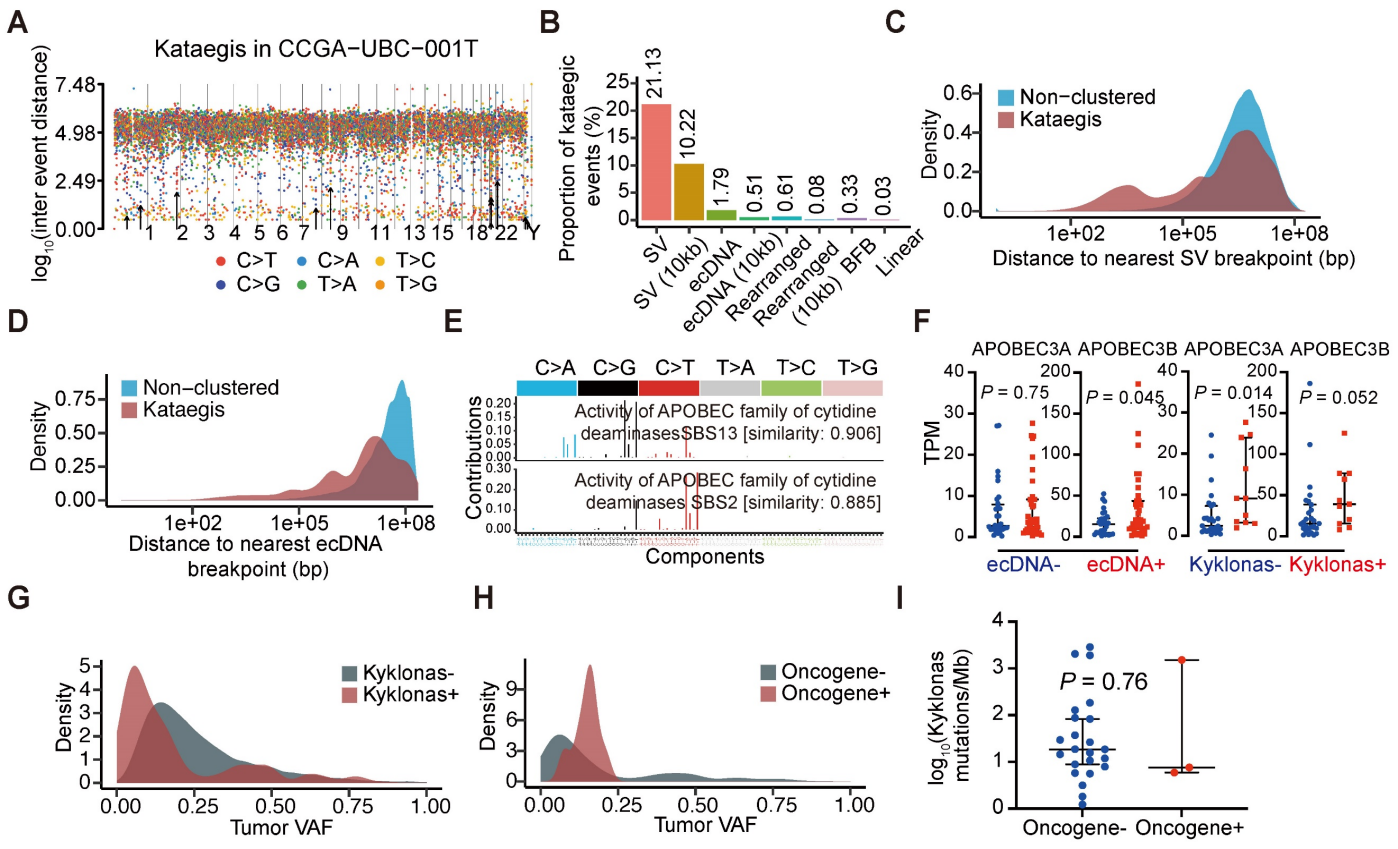
** Characteristics of hypermutations on ecDNAs.** (A) Rainfall plot illustrating the inter-mutation distances and the identified kataegis events (marked with black arrows) in sample CCGA-UBC-001T. (B) Proportions of kataegis events overlapping structural variants (SVs) and different types of focal amplifications. (C-D) Distance to the nearest SV (C) and ecDNA (D) breakpoints for non-clustered mutations and Kataegis mutations. (E) Mutational spectrum of kataegis on ecDNAs (kyklonas). (F) Comparison of the expression levels of APOBEC3A and APOBEC3B between ecDNA^-^ (n = 32) and ecDNA^+^ tumors (n = 38), and between tumors with (n = 11) and without (n = 27) kyklonas (Wilcoxon rank-sum test). (G-H) Distributions of the variant allele frequencies (VAFs) for non-ecDNA kataegis (Kyklonas^-^) and kyklonas^+^ (G), and kyklonic ecDNA with and without oncogenes (H). (I) Comparison of the kyklonas mutation burden between ecDNAs with (n = 3) and without (n = 23) oncogenes (Wilcoxon rank-sum test).

Clustered somatic mutations, such as kataegis, are frequently observed in cancer genomes 36. We detected kataegis in all UBC tumor samples (**Table S16**), demonstrating their prevalence in UBC. An example is shown with CCGA-UBC-001T (**Figure 5A**). Our analysis revealed that 21.13% of kataegis occurred on SVs while 10.22% were within 10 kb of SV breakpoints (**Figure 5B**). Unlike previous findings in glioblastoma or bone leiomyoma 37, only a small fraction of kataegis occurred on (1.79%) or near ecDNA (0.51% within 10 kb of breakpoint) in UBC samples (**Figure 5B**), highlighting the complexity of genomic alterations across different cancers. Compared to non-clustered mutations, a remarkably higher proportion of kataegis occurred near SV or ecDNA breakpoints (**Figure 5C and 5D**), and to a lesser extent near the BFB, Linear, or Heavily-rearranged amplification (**Figure S6A**). A tri-modal distribution of distances between kataegis and the nearest structural variations breakpoints was observed (**Figure 5C**), consistent with findings from a recent pan-cancer study 37. Kataegis were characterized by APOBEC3 signature (SBS2) and clock-like signature (SBS5) (**Figures S6B and S6C**). The underlying mechanism of SBS5 generation is largely unknown, but there is evidence that across multiple cancer types, APOBEC3 could drive the evolution of ecDNA 37, 38.

To further explore the relationship between ecDNA and APOBEC-mediated clustered hypermutation (kataegis) in UBC, we examined the presence of kataegis on the ecDNA region or within 10 kb of ecDNA breakpoints. Kyklonas (Greek for cyclone), referring to ecDNA-related kataegis, was found in 26.7% (12/45) of ecDNA^+^ tumors. These kataegis were dominated by the APOBEC3 signature (SBS2 and SBS13) (**Figure 5E**) and the APOBEC3 signature was also observed in kataegis within 10 kb of ecDNA breakpoints (**Figure S6D**). *APOBEC3A* and *APOBEC3B* are regarded as the two predominant APOBEC enzymes related to chromosomal mutagenesis 39. Moreover, compared to ecDNA^-^ tumors, an increase in *APOBEC3B* mRNA expression, but not *APOBEC3A* was observed in ecDNA^+^ tumors (Wilcoxon rank-sum test; *APOBEC3A*, *P* = 0.75; *APOBEC3B*, *P* = 0.045) (**Figure 5F**). Differences in the mRNA expression of *APOBEC3A* (Wilcoxon rank-sum test,* P* = 0.014) were also observed between tumors with and without kyklonic mutations (**Figure 5F**), indicating that *APOBEC3A* has an important role in the clustered mutagenesis of ecDNA. The variant allele frequencies (VAFs) of kyklonas tended to be lower than those of other kataegis (kyklonas^-^) (**Figure 5G**). Kyklonic events with higher VAFs were more often found on ecDNA with oncogenes, suggesting positive selection of oncogenes (**Figure 5H**). However, no difference in kyklonas mutation burden was observed between ecDNA with and without oncogenes (**Figure 5I**).

Chromothripsis is a catastrophic chromosomal shattering phenomenon involving multiple double-strand breaks, where many chromosomal fragments can be reassembled into a new derivative chromosome 40, 41. Several studies have suggested that chromothripsis could directly or indirectly create circular DNA fragments that can be subsequently amplified 40, 42-44. To examine the co-occurrence between chromothripsis and ecDNA, we inferred chromothripsis-like events by ShatterSeek software. We found that chromothripsis-like events are pervasive in UBCs, with 81% (65 out of 80) of tumor samples exhibiting such events (**Table S17**). Our analysis showed that chromothripsis can co-localize with ecDNA, with 84% of ecDNA^+^ tumors displaying chromothripsis-like events. Additionally, we detected chromothripsis-like events in 12% of ecDNA regions (**Figure S6E**). These data suggest that chromothripsis could be a contributing factor to the generation of ecDNA in UBC.

### Genetic background associated with ecDNA/eccDNA formation

**Figure 6:**
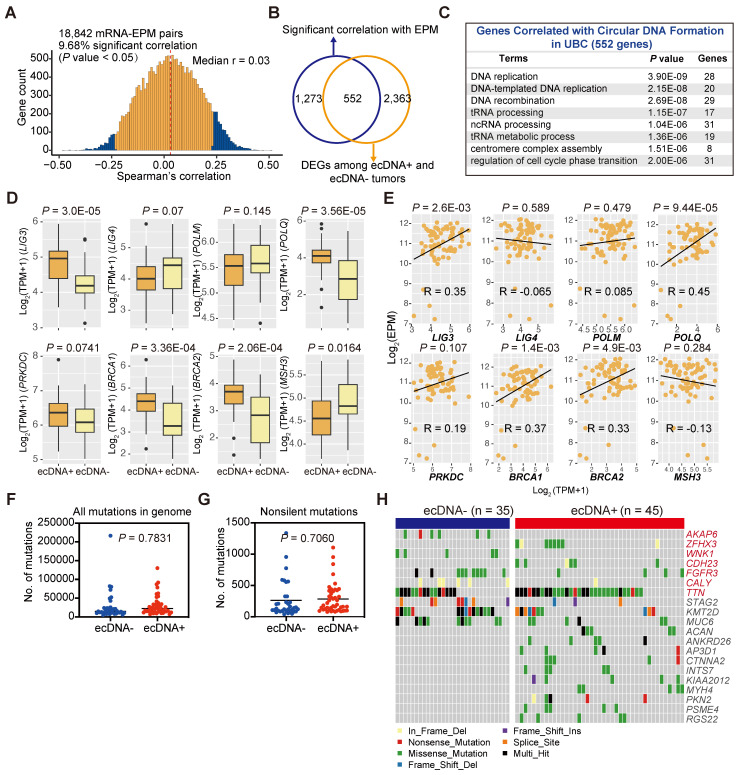
** Genetic background associated with ecDNA/eccDNA formation.** (A) Correlation between gene expression levels and the EPM in the UBC tumor samples (Spearman correlation test). (B) Overlap of genes significantly correlated with the EPM and genes differentially expressed between ecDNA^+^ and ecDNA^-^ UBC tumors. (C) Pathway enrichment analysis of genes correlated with ecDNA/eccDNA formation in UBC. (D) Expression levels of genes related to DNA repair and replication in ecDNA^-^ and ecDNA^+^ tumors (Wilcoxon rank-sum test). (E) Correlation analysis between the expression level of genes related to DNA repair and replication and the EPM in 80 UBC tumor samples (Pearson correlation test). (F-G) Comparison of the mutation load in the whole genome and exonic regions between ecDNA^-^ (n = 35) and ecDNA^+^ (n = 45) tumors (Wilcoxon rank-sum test). (H) Gene alterations in ecDNA^-^ and ecDNA^+^ tumors. Genes with significant differences in mutation frequency between ecDNA^-^ (n = 35) and ecDNA^+^ (n = 45) tumors (Fisher's exact test, *P* < 0.05) are marked in red.

DNA damage and repair during cell cycles can accumulate mutations in the cancer genome, and DNA damage has long been recognized as a critical event in the formation of ecDNA/eccDNA 45-48. Multiple hypotheses exist regarding the origins of ecDNA/eccDNA, often involving chromosome DNA damage and different endogenous repair systems such as mismatch repair (MMR), homologous recombination (HR), non-homologous end joining, and microhomology-mediated end joining (MMEJ) 45, 46. However, whether this correlation holds in solid tumors has never been thoroughly investigated. To identify genes potentially associated with the formation of ecDNA/eccDNA in cancers, we performed a correlation analysis between the gene expression levels and eccDNA levels of each tumor sample and examined the transcriptome of ecDNA^+^ and ecDNA^-^ UBCs (**Figures 6A and 6B**). Our data revealed that the expression level of 1,825 genes was correlated with eccDNA level, and 2,915 DEGs were identified between ecDNA^+^ and ecDNA^-^ UBCs (**Figure 6A; Tables S18, 19**). Of the significant correlations between eccDNA and expression, almost 30% (552 genes) overlapped with DEGs between ecDNA^+^ and ecDNA^-^ UBCs (**Figure 6B**). Pathway analysis of these 552 overlapping genes indicated enrichment in DNA replication and DNA recombination, as well as cell cycle signatures (**Figure 6C**). DNA repair genes have been demonstrated to be essential for ecDNA/eccDNA formation 45, 46. As shown in** Figure 6D**, the expression of *LIG3*, DNA Polymerase *POLQ,* and *BRCA1/2* was upregulated in the ecDNA^+^ tumors compared to ecDNA^-^ tumors (Wilcoxon rank-sum test; *LIG3*, *P* = 3.0E-05; *POLQ*, *P* =3.56E-05; *BRCA1*, *P* = 3.36E-04; *BRCA2*, *P* = 2.06E-04). Further analysis showed that the expression of *LIG3, POLQ* and *BRCA1/2* was significantly correlated with the eccDNA levels (*LIG3*, Pearson's R = 0.35, *P* = 2.6E-03; *POLQ*, Pearson's R = 0.45, *P* = 9.44E-05; *BRCA1*, Pearson's R = 0.37, *P* = 1.4E-03; *BRCA2*, Pearson's R = 0.33, *P* =4.9E-03) (**Figure 6E**). These findings suggest that DNA repair mechanisms play a role in the generation of ecDNA/eccDNA in UBCs.

We subsequently investigated somatic mutations in ecDNA^+^ and ecDNA^-^ UBCs to identify potential genetic contributors to ecDNA formation. Overall, the mutation load did not differ significantly between the two groups (Wilcoxon rank-sum test; all mutations in the genome, *P* = 0.7831; non-silent mutations, *P* = 0.7060) (**Figures 6F and 6G**), with similar findings observed in most cancer types from the TCGA datasets (**Figure S7A**). However, we identified several genes with differential mutation frequency between groups, including *ZFHX3*, *CDH23*, and *TTN*, which were preferentially mutated in ecDNA^+^ UBCs (**Figures 6H, S7B, and S7C**). Conversely, *AKAP6*, *WNK1*, *FGFR3*, and *CALY* were less frequently mutated in the ecDNA^+^ group (Fisher's exact test, *P* < 0.05). Additionally, some genes are equally mutated in both ecDNA^+^ and ecDNA^-^ groups (**Figure 6H**). These data suggest that certain genetic contributors may play a role in the formation or maintenance of ecDNA in UBCs.

### ecDNA/eccDNA and clinical features

**Figure 7:**
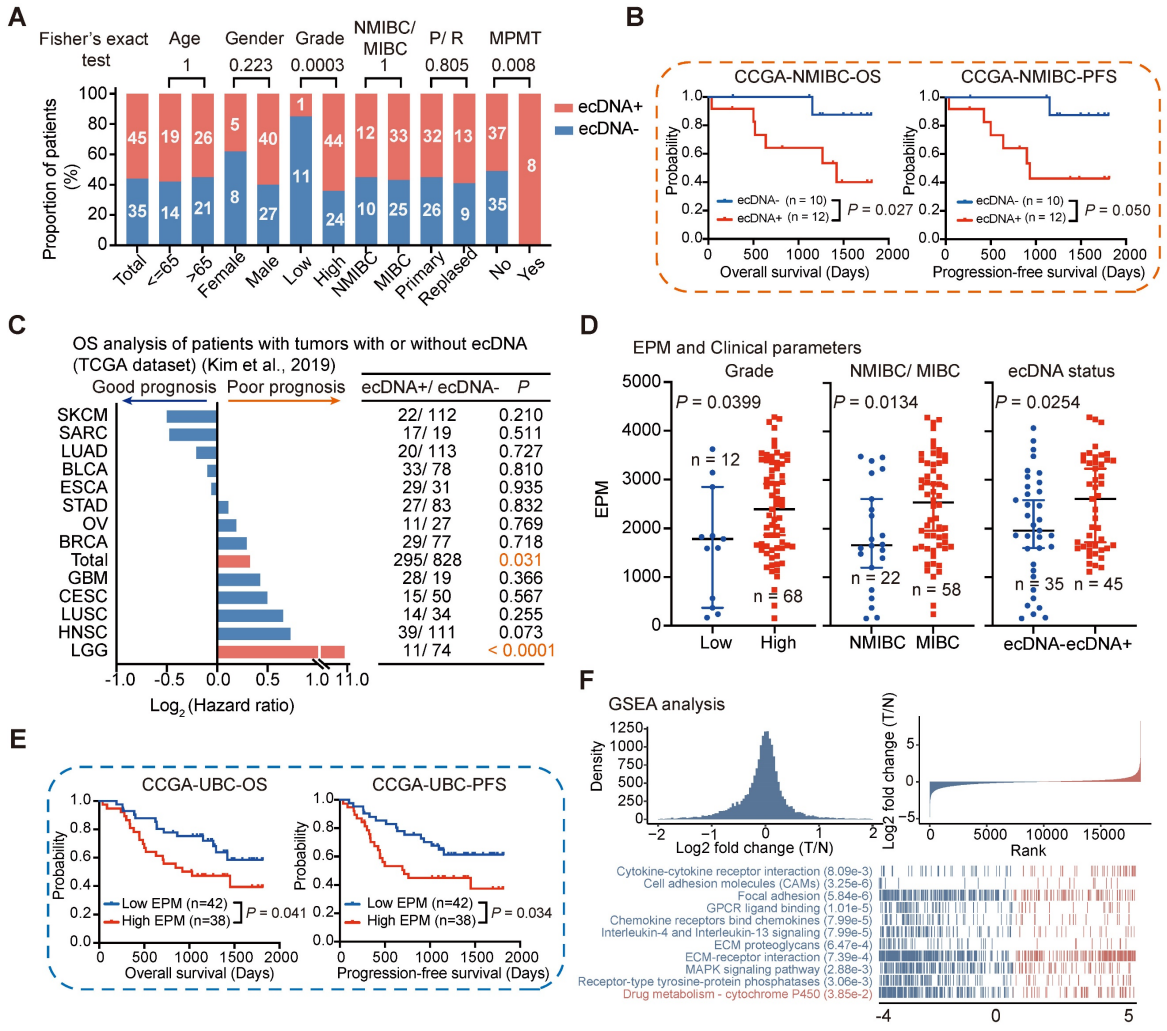
** Association of ecDNA/eccDNA and clinical features.** (A) Comparison of ecDNA frequency across different clinical groups (Fisher's exact test). (B) Survival analysis for CCGA-NMIBC patients with tumors with (n = 12) or without (n = 10) ecDNA (log-rank test). (C) Overall survival (OS) analysis for patients with tumors with or without ecDNA from 13 cancer types in TCGA datasets (log-rank test). (D) Comparison of EPM values among different clinical groups (Wilcoxon rank-sum test). (E) Survival analysis for CCGA-UBC patients with tumors with low (n = 42) and high (n = 38) levels of eccDNA (log-rank test). The mean EPM value was used as the cutoff value to define the high and low EPM groups. (F) Gene Set Enrichment Analysis (GSEA) identified the pathways that were significantly enriched in the EPM groups.

Tumors with ecDNA have been reported to exhibit aggressive biological features in some cancer types, such as high-risk medulloblastoma 16 and small-cell lung cancer 10. However, the role of ecDNA in UBC has not been previously studied. Therefore, we examined the association between ecDNA status and various clinical characteristics in UBC. Our analysis revealed that ecDNA was enriched in high-grade tumors and tumors from patients with multiple primary malignant tumors (MPMT). However, there was no significant difference in ecDNA distribution based on the depth of tumor invasion, age, or recurrence (Fisher's exact test; Age, *P* = 1; Gender, *P* = 0.223; Grade, *P* = 0.0003; NMIBC/MIBC, *P* = 1; Recurrence, *P* = 0.805; MPMT, *P* = 0.008) (**Figure 7A**).

We further investigated the relationship between ecDNA status and survival outcomes by categorizing UBC patients into two groups based on their ecDNA status. Interestingly, we found that ecDNA was associated with poor prognosis in patients with NMIBC (log-rank test; NMIBC, overall survival [OS]: *P* = 0.027, progression-free survival [PFS]: *P* = 0.050). However, the prognostic impact of ecDNA status on survival outcomes was not significant for MIBC patients (OS: *P* = 0.408, PFS: *P* = 0.344) (**Figures 7B and S7D**). This might be due to the small sample size or the distinct clinical features of this subgroup. We also examined the correlation between ecDNA status and prognosis in 13 different cancer types (each with at least 10 ecDNA+ tumors) using the TCGA-ecDNA data previously published by Kim et al 22. We found that ecDNA alone is insufficient to identify a group of patients with poor prognosis in most human cancers, except in the case of Low-Grade Glioma (LGG) (**Figure 7C**). These results suggest that the correlation between ecDNA and prognosis may depend on the tumor type and clinical staging, highlighting the need for further validation in larger cohorts.

The elevated eccDNA level in UBC tumors prompted us to investigate the relationship between eccDNA levels and various clinical and molecular variables. In general, higher eccDNA levels were strongly correlated with higher tumor grades, more advanced pathological stages, and positive ecDNA status (Wilcoxon rank-sum test; high grade versus low grade, *P* = 0.0399; MIBC versus NMIBC, *P* = 0.0134; ecDNA^+^ versus ecDNA^-^, *P* = 0.0254) (**Figures 7D and S7E**). We then stratified UBC patients into low EPM and high EPM groups using the mean EPM value as a cutoff and found that eccDNA level is significantly correlated with patient survival (log-rank test; OS, *P* = 0.041; PFS, *P* = 0.034) (**Figure 7E**). Multivariate analysis showed that compared with most clinical variables, a higher eccDNA load tends to poor prognosis, although the statistic was non-significant (**Figure S8**). GSEA showed that the Drug metabolism-cytochrome P450 pathway was enriched in the high EPM group, while oncogenic and inflammatory signaling pathways, such as cell adhesion molecules (CAMs), focal adhesion, and IL-4 and IL-13 signaling, were enriched in the Low EPM group (**Figure 7F**). These findings further underscore the critical role of dysregulated eccDNA in UBC.

## Discussion

UBC, particularly muscle-invasive UBC, poses significant challenges in diagnosis and treatment due to its debilitating nature and high costs. Despite recent advances in targeted treatments including FGFR3 inhibitors, as well as immune therapy mainly targeting T cells (PD1/ PD-L1), response rates remain suboptimal 6-8. This underscores the critical need for a deeper molecular understanding of UBC to drive the development of innovative targeted therapies. Extrachromosomal circular DNAs, especially the large cancer-specific ecDNA, have long been recognized as one potential driver of oncogenesis 13, 14, 17, 22, 24. Additionally, small eccDNAs play roles in various cancer-related cellular functions 29, 31, 32. Thus, systematically deciphering the characteristics of extrachromosomal circular DNAs (ecDNA/eccDNA) in tumors may contribute to improving the clinical management of cancer patients. Here, we present a comprehensive ecDNA/eccDNA landscape in UBC patients using a multi-omics strategy.

Our data demonstrate that ecDNA is common in UBC tumors, which is consistent with a previous finding reported by Kim et al. across pan-cancer studies 22. However, we found significant differences in ecDNA frequency between the TCGA cohort and our cohort; this may be related to differences in clinical characteristics, tumor purity, and ethnicity of the patients. Additionally, the current methodological challenges in identifying ecDNA and BFB may also contribute to these differences 33. Notably, oncogenes were frequently identified on ecDNA, which, unlike HSR-like amplification, contributes to a higher expression level of oncogenes. ecDNA seemed to be more prevalent in patients with high-grade tumors or MPMT at initial diagnosis. NMIBC ecDNA^+^ patients showed worse survival compared to patients without ecDNA. However, a main limitation of our study is the relatively low number of NMIBC patients in our CCGA-UBC cohort. We also examined the correlation between ecDNA status and prognosis in 13 different cancer types in the TCGA datasets 22. We found that the prognostic significance of ecDNA status varies across different types of cancers and clinical stages. While ecDNA status is a potential marker of poor prognosis in NMIBC and LGG, it does not universally apply to all cancer types or stages. This underscores the complexity of cancer biology and the necessity for more extensive studies to validate these associations. Moreover, future studies could integrate single-cell sequencing with ecDNA analysis to identify specific malignant cell populations that harbor ecDNA and their associated gene expression profiles. This approach could also illuminate the functional roles of ecDNA in different cellular contexts and its contributions to tumor biology.

The detection of ecDNA in urine sediment samples from patients with ecDNA^+^ tumors is noteworthy. While detecting ecDNA in cancer cells and tumor tissues is relatively straightforward due to their high and enriched copies, identifying ecDNA in urine samples is unexpected given the anticipated dilution of ecDNA in such samples. The high detection rate (74%) underscores the potential of this method for non-invasive cancer-specific ecDNA monitoring. The ability of low-depth WGS to detect ecDNA in most urine samples indicates a highly sensitive detection approach. However, sample quality, including the time of collection, handling, and storage conditions, must be standardized to ensure reliable detection 49. The time-matched collection of urine and tumor samples likely contributed to the high concordance observed between urine and tumor ecDNA profiles. Furthermore, the observation that ecDNA profiles in urine were entirely different from those in tumor tissues for two patients highlights the issue of intratumoral heterogeneity, suggesting that urine-derived ecDNA might reflect different subclonal populations within the tumor. This presents a promising non-invasive method for cancer diagnosis and monitoring, warranting further research to optimize detection methods, understand the impact of sample quality, and explore the clinical implications of intratumoral heterogeneity reflected in urine-derived ecDNA.

Currently, metaphase-FISH serves as the gold standard for detecting ecDNA. This method allows a clear determination of amplification outside the chromosomes. However, clinical samples primarily comprise formalin-fixed, paraffin-embedded (FFPE) specimens, restricting analysis to interphase FISH. Therefore, developing image algorithms to recognize ecDNA based on interphase FISH would be advantageous for widespread clinical use. Additionally, given the critical role of ecDNA in tumors, targeting pathways involved in its formation and maintenance may prove effective in cancer therapy 25.

Research on small non-amplified eccDNA has advanced significantly in recent years 15, 18, 29, 50; however, the genome-wide distribution and features of eccDNA in multiple cancer types such as UBC, remain poorly understood. By integrating Circle-Seq and Circle-Map^++^, we comprehensively characterized eccDNA in UBC, uncovering several findings: 1) eccDNA levels are significantly elevated in tumors compared to ATs, potentially due to DNA damage and repair cycles in cancer cells, 2) tumor-derived eccDNAs harbor a higher percentage of small circles, 3) many CAGs, such as CCR4, exhibit heightened eccDNA generation in UBC tissues compared to ATs, 4) genes exhibiting differential eccDNA prevalence between UBC tissues and ATs were notably enriched in cancer- and immune-related signatures, including inflammatory response, IL-17 signaling pathway, and TNF signaling pathway. Most importantly, we show that eccDNA levels are correlated with worse patient outcomes.

In line with previous studies 17, 50, we demonstrate that eccDNA formation is not random but preferentially occurs from GC-rich, repetitive, and protein-coding gene regions, often originating from mono-allelic sources. We identified a distinctive 'ladder' pattern in the size distribution of eccDNAs, where peaks are spaced approximately every 200 bp. This pattern is similar to linear DNA derived from apoptotic cells, suggesting the potential relationship between eccDNA formation and apoptosis 29. In our study, while the overall correlation between mRNA and eccDNA was generally weak, we did observe some significant positive correlations. However, we observed a notable difference in the number of strongly correlated mRNA-eccDNA pairs between tumor and AT samples. This discrepancy could be attributed to several factors. Tumors and ATs might have different eccDNA dynamics or biological contexts, which could influence the correlation patterns. Additionally, the biological processes in tumors, such as ongoing transcriptional changes or aberrations, might affect the interaction between mRNA and eccDNA differently compared to ATs. Moreover, genes or regulatory elements on eccDNA could be transcribed independently of canonical promoters 31. We found that tumor-derived eccDNAs are more likely to be generated from protein-coding gene regions. Although most of these gene-related circles are unlikely to be directly expressed in tumors, it is reasonable to suspect that they have transient effects on cancer phenotypes.

In summary, we systematically mapped the landscape of extrachromosomal circular DNA (ecDNA/eccDNA) in UBC and evaluated its potential impact on genome remodeling, gene expression, and patient outcomes. Additionally, we present the novel finding that cancer-specific ecDNA can be detected in urine samples for the first time. Our integrated profiling of extrachromosomal circular DNA not only enhances our understanding of the role of ecDNA/eccDNA in bladder cancer but also offers valuable data resources and bioinformatics tools for future research on cancer and extrachromosomal circular DNA.

## Materials and Methods

### Data reporting

A statistical method was not used to predefine the sample sizes. The experiments were not randomized and were not blindly analyzed.

### Clinical sample acquisition, preparation, and ethical permission

Paired tumors, non-tumor adjacent bladder tissues (ATs), and urine samples used in the present study were obtained from Yantai Yuhuangding Hospital (Shandong, China) for the current Cancer Circular Genome Atlas (CCGA) project. Patients (n = 80) who underwent radical cystectomy or TURBT from January 2017 to December 2021 were selected from the Yuhuangding hospital, and the UBC patients did not receive any anti-tumor therapy before surgery. The enrolled patients were designed as CCGA-UBC patients. To avoid the risk of inadvertent protected health information disclosures, the patient's name and medical record number were de-identified, and each sample was assigned a new research ID (CCGA-UBC-No. T/N/U). Each tissue sample was washed three times with phosphate buffer saline (PBS) immediately after surgery to remove blood. Paired NATs were taken at least 2 cm away from the tumor margin. The middle section of each tissue block was stained with hematoxylin and eosin (H&E) for tumor content and cellularity analysis, which was done by the pathology in the hospital. The tumor histology of each case was independently evaluated by two expert genitourinary pathologists (C.L. and G.Y. ). The remaining bladder tissues were ground in liquid nitrogen and then divided into several parts to reduce the impact of intra-tumor heterogeneity on trans-omics analysis. For each case, ~ 100 mg tissue sample was used for RNA extraction and RNA-Seq; ~ 30 mg tissue sample was used for DNA extraction, whole genome/exome sequencing (WGS/WES), and Circle-Seq. Approximately 50 ml of morning spot-urine samples added with 1 ml of 500 mM EDTA were collected on the day before surgery. To precipitate urinary sediment, the urine samples were spun 3000 x g at 4°C for 10 min. The urinary sediment was stored at -80°C for further experiments. A total of 80 CCGA-UBC patients were enrolled with the clinical information including age, gender, pathologic stage, grade, family history, date of surgical resection, recurrence status, date of the last visit, and survival status. Detailed clinical characteristics of our enrolled patients were recorded and included in the present study (**Figure S1C; Table S1**). All patients were observed for progression-free survival (PFS) and overall survival (OS). PFS was measured as the time from the first surgery to disease progression or death, and OS was measured as the interval between the first surgery and death. All enrolled patients were followed with a median time of 1,123 days and the final patient follow-up was in September 2022. All CCGA-UBC patients or their relatives consented to the use of their biomaterials for cancer research. Before the study initiation, the protocol was officially approved by the institutional review board (IRB) of the BGI-Shenzhen and Yantai Yuhuangding Hospital.

### High molecular weight genomic DNA (HMW *g*DNA) extraction

Urinary sediment samples, ~30 mg tumor, and AT samples were subjected to genomic DNA (gDNA) extraction using the MagAttract HMW DNA kit (Qiagen), according to the manufacturer's instructions. DNA quantification was performed using the Qubit Fluorometer (Invitrogen). DNA integrity was assessed on 0.8% agarose gel.

### WGS and WES

#### Library preparation and sequencing

About 0.5 µg of total gDNA of each sample was sonicated to a size range of 300-500 bp using the Covaris LE220 (Covaris). DNA fragments were then end-repaired, A-tailed, and adapter-ligated using the MGIEasy DNA Library Preparation Kit (MGI). The quality of each library, including size distribution and concentration, was assessed using the Agilent Bioanalyzer 2100 system. The constructed library was deep sequenced on the DNBSEQ-T1&T5 (PE 150) platform (BGI-Shenzhen). For each tumor sample and its ATs, WGS generated a mean coverage depth of 42X (range: 28-55X) and 40X (range: 28-48X) respectively. For urine samples, WGS generated a mean coverage depth of 11X (range: 9-15X) for further ecDNA detection.

About 0.7 µg gDNA per sample was used as input material for WES library preparation. WES library preparation was performed with the MGIEasy Exome Capture V4 Probe following the manufacturer's instructions. Pooled libraries were sequenced on the DNBSEQ-T1&T5 (PE 100) platform (BGI-Shenzhen). We obtained a mean sequence coverage depth of 160X (range: 102-219X) and 83X (range: 44-123X) for each tumor and AT sample respectively.

#### Data preprocessing and alignment

FastQC v0.11.3 was conducted to qualify the raw sequencing reads. The low-quality reads were trimmed as clean reads using the fastp v0.19.6 51 software with default parameters. The remaining high-quality clean reads were mapped to the human reference genome (GRCh38) using BWA-MEM v0.7.17 52 and duplicate reads were marked using GATK MarkDuplicate (GATK v4.2.2.0). After alignment and trimming, all bam files were sorted and indexed using SAMtools v1.15 53 for downstream processing analyses, including somatic copy number alteration (SCNA) and ecDNA analysis, somatic mutation, and structural variation detection.

#### Somatic mutation calling, filtering, and annotation

Somatic mutations, including single nucleotide variants (SNVs) and insertions/deletions (Indels), were identified via local assembly of haplotypes by Mutect2 54, with the tumor and matched normal BAMs as inputs. Analysis of WGS and WES data was performed separately. FilterMutectCalls in GATK (v4.2.5.0) was used to filter the raw output of Mutect2. ANNOVAR (Version 2020.06.07) 55 was used to annotate SNVs and Indels. Next, the results of WGS and WES were merged into a final call set for downstream analysis.

#### Mutation significance analysis

Significantly mutated genes (SMGs), harboring significantly more non-synonymous mutations than the background, were identified using MutSigCV (1.41) 56 with q-values < 0.1. A total of 24 SMGs were identified in this study.

#### Mutational signature analysis

The SNVs, including non-synonymous and synonymous mutations, were classified into 96 substitution types, based on six base substitutions (C > A, C > G, C > T, T > A, T > C, and T > G) and neighboring bases. A non-negative matrix factorization (NMF) approach was used for mutation signature discovery. Then the signatures were compared to known signatures from the Catalogue of Somatic Mutations in Cancer (COSMIC) database 57 and cosine similarity was calculated.

#### Tumor mutational burden (TMB)

The TMB was calculated with the total number of mutations (SNVs and Indels) counted divided by the length of the coding sequence region. Only the non-synonymous mutations, including frameshift insertion, frameshift deletion, non-frameshift insertion, non-frameshift deletion, missense, nonsense, nonstop, start-loss, and splice site, were used for TMB estimation.

#### Kataegis analysis

Kataegis were defined as those segments harboring six or more consecutive SNVs with an average inter-mutation distance <= 1000bp (**Table S16**).

#### Somatic copy number alteration (SCNA) analysis

The tumor-specific SCNAs were calculated from paired tumor-AT WGS data using CNVkit (v0.9.3) 58 with default settings. To identify significantly deleted or amplified regions across all samples, Genomic Identification of Significant Targets in Cancer (GISTIC2) 59 was performed. A log2 ratio above 0.1 was considered a “gain” and a log2 ratio below -0.1 was considered a “loss”. The copy number data was prepared for further ecDNA detection.

#### Detection of focal amplifications with AmpliconArchitect

Aligned WGS sequences and genomic segments with copy number (CN) of more than 4.3 copies and with lengths larger than 10 kb (seed regions) were used as input data for AmpliconArchitect (AA) 33 to infer the architecture of amplicons. AA then searches for other regions that belong to the amplicon by exploring the seed intervals and extends beyond the intervals if it encounters CN changes or discordant edges that support a breakpoint. After the collection of intervals and breakpoints, a breakpoint graph and a simple cycle were formed separately. The detected amplicons were annotated with the NCBI RefSeqGene database (GRCh38). AmpliconClassifier (AC) (version 0.4.9, https://github.com/jluebeck/AmpliconClassifier) 22 was then performed to classify the AA output data into different types of focal amplifications (including BFB, Circular, Heavily-rearranged, and Linear) and to extract coordinates of the genomic regions corresponding to those amplicons. When a patient presents with multiple amplification topologies, we classify them according to the following priority order: ecDNA, BFB, Heavily-rearranged, and linear amplifications.

#### Cancer-associated genes (CAGs)

A list of CAGs, including oncogenes, tumor suppressor genes (TSGs), and some cancer-driver genes, was compiled from COSMIC databases, and a previous study 60.

#### Comparisons of focal amplifications between TCGA-BLCA and CCGA-UBC cohorts

The focal amplification data call by AA from The Cancer Genome Atlas BLCA datasets (TCGA-BLCA) were downloaded from a previous study 22. The frequency of focal amplifications and the most frequent oncogenes carried on focal amplifications within the TCGA-BLCA cohort and CCGA-UBC cohort were compared.

#### Inference of chromothripsis

Somatic structural variations (SVs) were identified on tumor samples using the Delly2 (v1.1.3) 61 and Manta (v1.6.0) 62 software by taking paired ATs as control, and the final list of SVs is merged from the output data of Delly and Manta. Based on the merged SV and CNA results, chromothripsis-like patterns were identified and visualized by Shatterseek (v0.4) with the default settings 47. Focal amplification region and segments from chromothripsis were overlapped using the bedtools intersect command 22. The focal amplifications and eccDNA were labeled as overlapping with chromothripsis with their overlapping regions longer than 1 bp.

### Circle-Seq

#### Circular DNA purification and enrichment

Circular DNAs were isolated from tumor and AT samples using the Circle-Seq workflow as previously described with slight modifications 18. Firstly, to digest the non-circularized DNA, one microgram of total HMW *g*DNA from each sample was treated with exonuclease V (NEB) for 144h at 37°C, adding additional fresh reaction buffer, ATP, and 30 units of exonuclease V every 24h. After digestion for 144h, exonuclease V was inactivated for 30 min by heating at 70°C. The reaction mixes were then purified with 2X DNA clean beads (Vazyme) and dissolved in 50 µl of nuclease-free water. Successful removal of linear chromosomal DNA was confirmed by COX5b PCR (30 cycles) with reverse primer 5' AGTCGCCTGCTCTTCATCAG 3' and forward primer 5' GGGCACCATTTTCCTTGATCAT 3'. The target band of PCR products is around 100 bp (not shown). Notably, the complete elimination of linear chromosomal DNA by exonuclease alone is challenging due to the potential presence of various complex structures or modifications, such as cross-links, cruciforms, oxidation, and G-quadruplexes.

#### Rolling circle amplification (RCA) of circular DNA

To increase the signal, 12 µl of the enriched traces number of circular DNA was used as the template for RCA. The 40 µl reaction system involved 14.2 ul RNase-free water, 0.8 µl 100mM DTT, 4 µl 2.5 µM dNTP mixture (Takara), 2 µl exonuclease-resistant random primer (Thermo), 4 µl 10× Phi29 buffer (Thermo) and 1 µl Phi29 polymerase (Thermo), and were incubated at 30°C for 48h. The reaction was stopped by heat-inactivation at 65C for 10 min. All Circle-Seq experiments were performed by the same experimenter (W.L.) to avoid potentially biased interpretation.

#### Circular DNA sequencing (PE 150)

phi29-amplified DNA samples were performed library construction and sequencing using the same experimental procedures of WGS. Circle-Seq produced an average of 114 and 124 million high-quality reads for each tumor and AT sample respectively (**Table S2**).

#### Comparison of the performance between Circle-Map and Circle-Map^++^

We have rewritten and modified the Circle-Map [34]software using the C++ language to improve the computation performance (hereafter noted as Circle-Map^++^). To evaluate the efficiency between Circle-Map and Circle-Map^++^, we generated 2 X 5,423,377,089 PE reads (corresponding to 508,926 eccDNAs) using the Circle-Map simulate function. We then used Circle-Map and Circle-Map^++^ to detect eccDNA from the simulated data in the same computing environment. The capture rate, accuracy, running time, and memory usage were calculated and compared using R and bedtools.

#### Identification of circular DNA from short sequence reads with Circle-Map^++^

Sequencing FASTQ files were processed to BAM files by the same pipeline as we previously described 50. Circle-Map^++^ was then conducted to identify circular DNAs from sequencing data. Several filtering steps were performed to obtain a robust set of eccDNAs from the short-read sequencing dataset: (1) split reads ≥ 2, (2) Circle score ≥ 200, (3) Coverage increase in the start coordinate ≥ 0.33, (4) Coverage increase in the end coordinate ≥ 0.33, (5) Coverage continuity ≤ 0.1, (6) The SD of coverage smaller than the mean coverage over the whole eccDNA region. To eliminate the influence of false-positive of supper longer eccDNA caused by the same read containing both split and discordant reads, we filtered circles (≥ 2 kb) without a discordant read.

#### Repetitive DNA quantification

The number of reads that were aligned to repeat regions (RepeatMasker open-4.0.5; http://repeatmasker.org) was counted by BedTools multicov.

#### GC content in circular DNA

The GC content was calculated from the reference sequence of all detected circularized regions. We used the mean G+C content for statistics.

#### Generation of random in silico eccDNAs

To better understand the genomic features of eccDNA from tumors and ATs, we generated randomized regions (*In Silico* eccDNAs) across the genome using the BedTools shuffle command as baseline information. The length distribution and the number of *in silico* eccDNA were consistent with circular DNAs detected in ATs. The GC content, eccDNA mapped to genes, and reads aligned to repeats of *In Silico* eccDNAs were computed.

#### Differential eccDNA abundance analysis

Comprehensive gene annotation information was derived from Ensembl BioMart (Ensembl Genes 107, GRCh38.p13). Overlaps between coding genes and eccDNA were performed using BEDTools intersect (Bedtools v2.30.0). For the purpose of quantification, the relative eccDNA abundance on each gene was calculated according to the following formula:



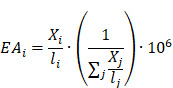



Where 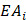 represents the eccDNA abundance of a specific gene, 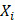 and 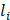 represent the unique eccDNA junction counts (here we use the start site of eccDNA), and the length for a specific gene, 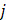 represents the last gene counted.

To identify genes with differential eccDNA abundance between the tumors and matched ATs, a Wilcoxon rank-Sum test was performed; The genes with absolute log2 fold change > 0.5 and* P* < 0.01 were considered statistically significant.

#### B-allele analysis

We use alleleCount v4.2.1 on all bam files to get the allelic counts using the pre-collected database from ASCAT (v3.0) 63. B-allele frequency was obtained by using ascat.prepareHTS.

#### Validation of eccDNA recordings

Junction sequences of three typical *CCR4*-related circles were verified by outward PCR and Sanger sequencing. Outward-directing PCR oligos were designed in Snapgene (V3.1.4) and listed in **Table S10**. PCR was done with NEBNext High-Fidelity 2X PCR Master Mix according to the manufacturer's instructions. phi29 amplified samples were used as templates. The PCR products were tested by agarose (2.5%) gel electrophoresis, and the target products were recovered by QIAEX II Gel Extraction Kit. To confirm the junction site of all selected eccDNAs, Sanger sequencing was performed on target products.

#### Detection of circular DNA from long reads

We re-sequenced 9 paired tumor-AT samples (CCGA-UBC-005/ 013/ 015/ 016/ 017/ 021/029/037/050) with long-read sequencing technology (PacBio). T7 endonuclease I (NEB) was used to debranch the RCA products of these samples. Then, 8 µg of debranched-RCA products were sheared using g-Tubes (Covaris) and concentrated with AMPure PB magnetic beads. Each SMRT bell library was constructed using the Pacific Biosciences SMRT bell. The constructed library was size-selected by Sage ELF for molecules 8-12 kb, followed by primer annealing and the binding of SMRT bell templates to polymerases with the DNA Polymerase Binding Kit. Sequencing was carried out on the Pacific Bioscience Sequel II platform for 30 h Pacific Bioscience Sequel II platform for 30 h. The PacBio long-read sequencing generated an average of 157,084 and 62,295 high-quality reads per tumor and AT sample respectively.

#### Generate HiFi reads and eccDNA assembly

The subreads generated by Pacbio Sequel II were fed to CCS v6.4.0 for generating Highly Accurate Single-Molecule Consensus Reads (HiFi Reads). HiFi reads were then aligned to GRCh38 using minimap2 v2.24 64 with the following parameters: -x map-HiFi -c—secondary=no -t 10. The alignment for each read was stored in PAF format. To obtain the consensus boundary and sequence of each eccDNA from the mapped RCA long reads, we use the eccDNA_RCA_nanopore (https://github.com/YiZhang-lab/eccDNA_RCA_nanopore) to get the eccDNA fragment composition 29. Only eccDNAs with at least two passes were kept for downstream analysis. The Ribbon (https://genomeribbon.com/) was performed to visualize the assembled sequences of the eccDNA from multiple fragments.

### RNA sequencing

#### RNA-extraction and RNA-Seq

Total RNA was extracted from frozen tissue specimens by using TRIzol Regent (Thermo), and RNA integrity and concentration were assessed in an Agilent 2100 Bioanalyzer (Agilent). Only specimens with RNA integrity number (RIN) > 5.0 were subjected to the following RNA-Seq library preparation. The libraries were prepared using MGIEasy RNA Library Prep Kit (MGI-BGI) with 500 ng total RNA as input and sequenced on the DNBSEQ-T1&T5 (PE 100) platform (BGI-Shenzhen). RNA-Seq produced an average of 385 and 317 million high-quality reads for each tumor and AT sample respectively (**Table S2**).

#### RNA-Seq data analysis

FastQC v0.11.3 was conducted to assess the RNA-Seq data quality. Low-sequencing data were trimmed using fastp (v0.19.6) 51. Processed read pairs were aligned to the human reference genome (GRCh38) using subjunc aligner (subread v2.0.1) 65. Mapped read counts per gene were measured using featureCounts (subread v2.0.1) and GENCODE version 40 (Ensembl 106) gene annotation. Read counts per gene were determined using the featureCounts software. Raw read counts were normalized and further analyzed using the DESeq2 R/Bioconductor package 66.

### mRNA-eccDNA correlation in tumors and ATs

The correlation between eccDNA abundance and mRNA expression for each gene in tumors or ATs was measured using the Spearman correlation coefficient. The correlation coefficient and *P* value were reported. Only genes with transcript per million (TPM) > 5 in each sample were used for mRNA-eccDNA correlation analysis.

### Pathway enrichment analysis

ClusterProfiler v4.4.4 67 R package was conducted for Gene-Set Enrichment Analysis (GSEA), Go-term, and KEGG pathway analysis.

### Survival analysis

Pan cancer survival data were obtained directly from the TCGA Data Portal (https://tcga-data.nci.nih.gov/tcga/). Kaplan-Meier (KM) curves and log-rank statistics were used to examine the progression-free survival (PFS) or overall survival (OS) of the patients in different clinical or molecular subgroups. Kaplan-Meier survival curves were plotted using GraphPad and hazard ratios (HR) were computed using Cox proportional hazards regression analysis.

### Interphase FISH

Interphase FISH was conducted using the ZytoLight ® SPEC CCND1/CEN 11 Dual Color Probe to conform the amplification status of *CCND1* in four available FFPE tumor samples. Briefly, FFPE samples were deparaffinized in xylene, rehydrated through ethanol washes, and rinsed in distilled water. This is followed by Heat-induced epitope retrieval and protein digestion. Slides were then dehydrated by washing in 70%, 85%, and 100% cold ethanol stored at -20 °C (60s in each solution). FISH probes, diluted in hybridization buffer, were applied to the slides and covered with a coverslip. Denaturation of the slides occurred at 72 °C for 5 minutes, followed by an overnight (14-18h) hybridization at 37 °C. Slides were washed within 2×SSC/0.3%NP-40 (pH 7.0-7.5). The dried slides were stained with 10μl of DAPI buffer. Images were taken using OLYMPUS BX53.

### Quantification and statistical analysis

The statistical tests used to analyze the data are described in the main text and figure legends. Statistical tests including Cox proportional hazard analysis, log-rank test, Fisher's exact test, Wilcoxon rank-sum test, Pearson or Spearman correlation, and t-test were performed with R or GraphPad Prism. Differences between groups for continuous variables were made with the Wilcoxon rank sum test or paired t-test. Comparisons of categorical variables were investigated by Fisher's exact test. Pearson or Spearman correlation was used to study the correlation between continuous variables. Survival curves (log-rank test) were used to assess OS and PFS. All statistical evaluations were two-sided and were carried out by researchers and independent statisticians, and *P* < 0.05 indicated statistical significance.

## Supplementary Material

Supplementary figures.

Supplementary tables.

## Figures and Tables

**Table 1:** Clinical characteristics of the CCGA-UBC cohort.

Category	UBC
**No. of patients**	80
**Age, yr-no. (%)**	
≤65	33 (41)
>65	47 (59)
**Gender-no. (%)**	
Male	67 (84)
Female	13 (16)
**Smoking status-no. (%)**	
Yes	34 (43)
Never	45 (56)
NA	1 (1)
**Histologic grading-no. (%)**	
Low	12 (15)
High	68 (85)
**T-category-no. (%)**	
MIBC	58 (73)
NMIBC	22 (28)
**T-stage-no. (%)**	
T1	22 (27)
T2	30 (38)
T3	19 (24)
T4	9 (11)
**Primary/ Relapesd-no. (%)**	
Primary	58 (73)
Relapesd	22 (28)
**Surgical approach-no. (%)**	
Transurethral resection of bladder tumor	6 (8)
Radical cystectomy	70 (88)
Partial cystectomy	4 (5.0)
