# Supplementary material for: Extrachromosomal circular DNA orchestrates genome heterogeneity in urothelial bladder carcinoma
Source: Theranostics. 2024 Aug 19;14(13):5102–22. doi: 10.7150/thno.99563 (PMC11388072; doi:10.7150/thno.99563)
Supplement: Supplementary file 1 — Supplementary figures. [file thnov14p5102s1.pdf]

This file includes: Supplementary Figure S1-S8

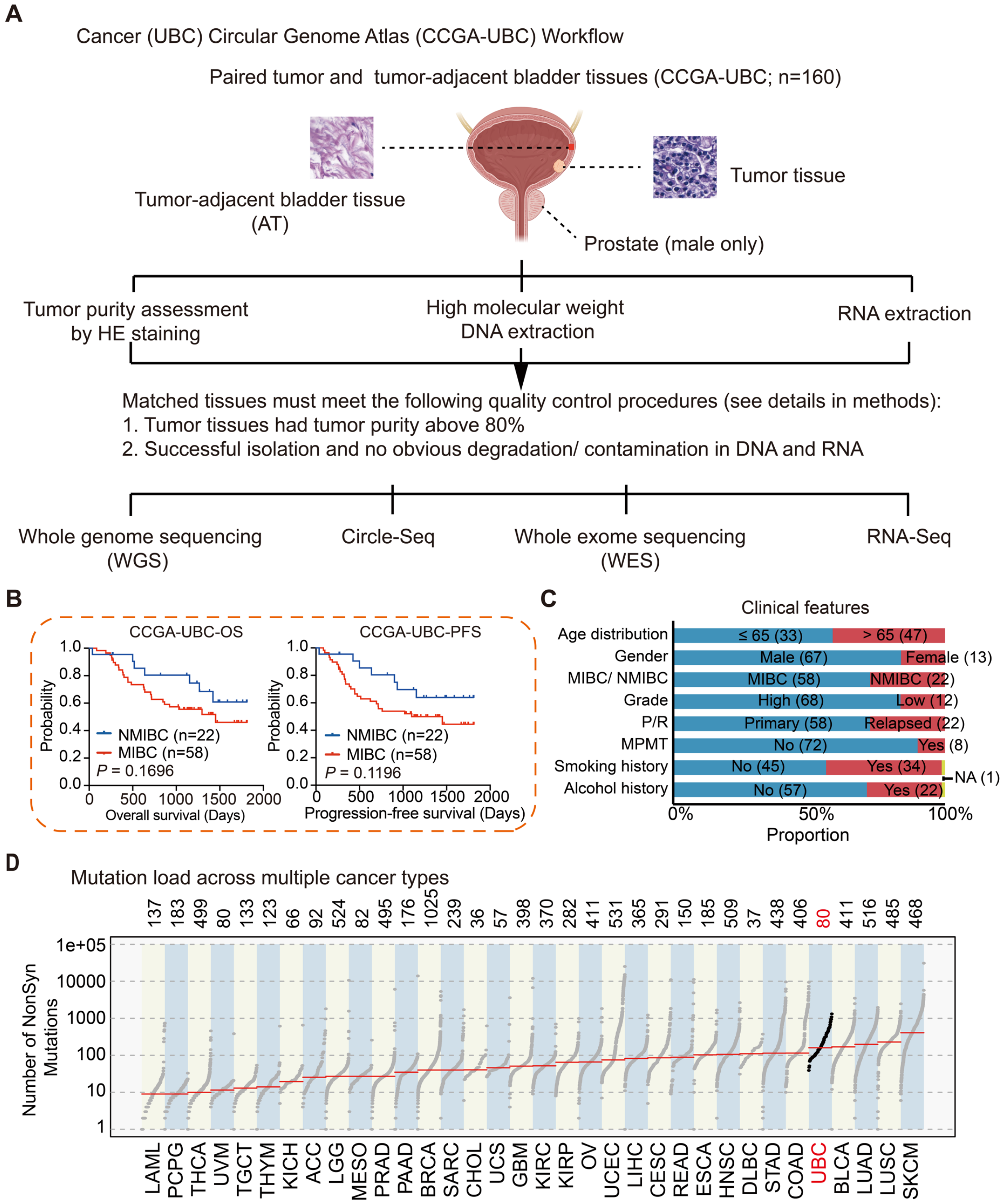

Figure S1. The experimental workflow of the CCGA-UBC study and the clinical characteristics of the patients (n = 80)

(A) A total of 80 pairs of freshly snap-frozen tumor tissues and Adjacent Tumor Tissues (ATs) were collected from UBC patients. Hematoxylin-Eosin (HE) staining was performed to assess tumor purity and pathology. Sample filtering was performed based on the following criteria: 1) Tumor tissues with tumor purity above 80%; 2) Successful isolation of high molecular weight genomic DNA (HMW gDNA) and qualified RNA (RNA integrity number > 5) from both tumor and AT samples.

(B) Survival analysis of patients with MIBC (n = 58) and NMIBC (n = 22) (log-rank test).
(C) Summary of clinical characteristics of 80 CCGA-UBC patients. Clinical features include age, gender, MIBC/NMIBC status, grade, primary/relapsed tumors, presence of f multiple primary malignant tumors (MPMT), smoking history, and alcohol history.
(D) Pan-cancer distribution of non-synonymous mutation counts. Each dot represents a tumor sample from the TCGA datasets or the CCGA-UBC cohort. Red bars indicate the median number of non-synonymous mutations for each cancer type. The sample size for each tumor type is labeled above the graph.

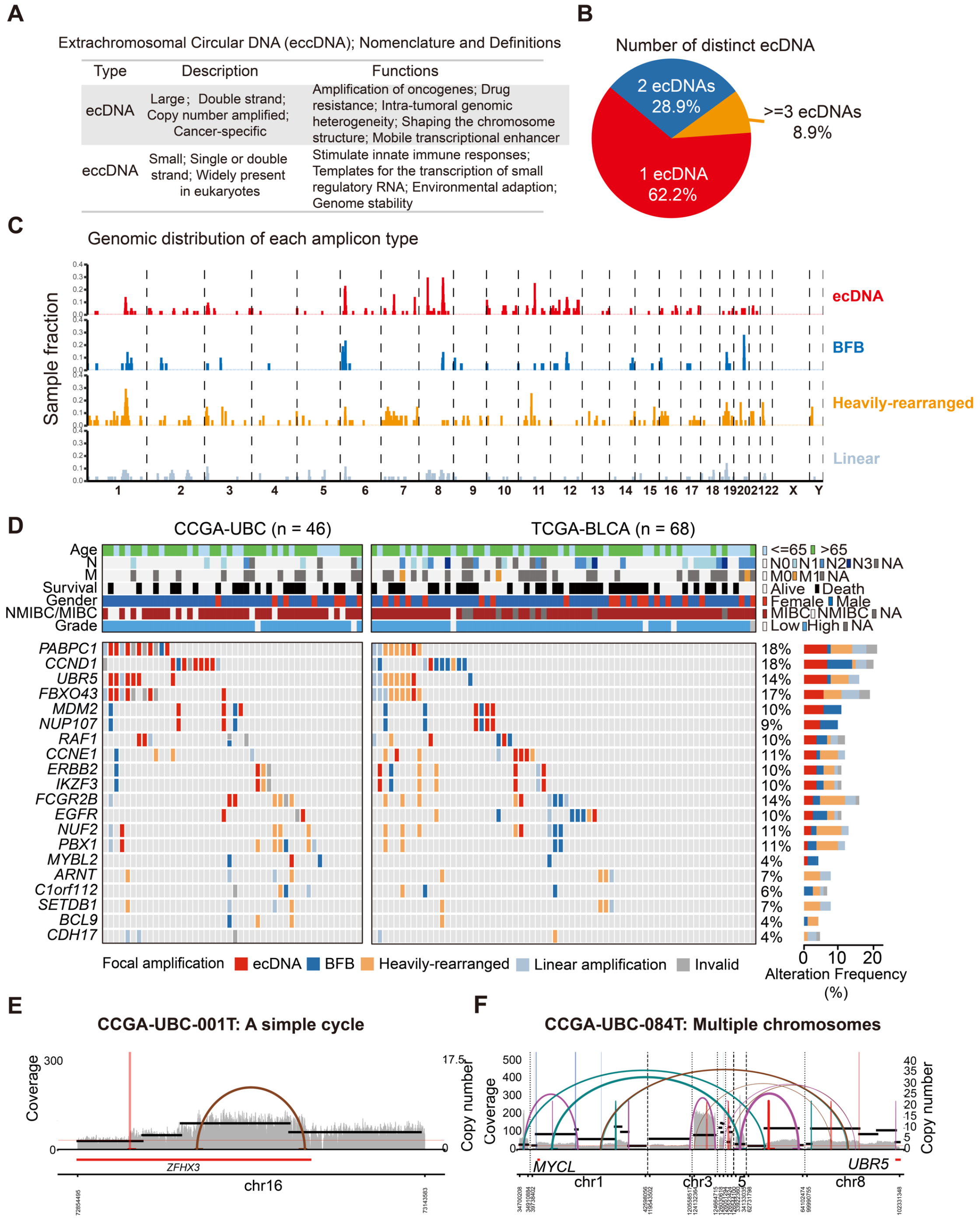

Figure S2. Focal amplification landscape of CCGA-UBC Samples

(A) Summary of the definitions and functions of two main types of extrachromosomal circular DNA (ecDNA and eccDNA).

(B) Pie chart showing the percentages of tumors with different numbers of ecDNA species among the total 45 ecDNA^{+} cases.

(C) Genomic distribution of each amplicon class, including ecDNA, BFB, Heavily-rearranged, and Linear.

(D) Oncogene profile and associated clinicopathologic features of patients with any type of focal amplification (carrying oncogenes) in the CCGA-UBC cohort (n = 46) and TCGA-BLCA cohort (n = 68).

(E-F) AA-generated structural variant (SV) and breakpoint graph of examples of a simple cycle (E) and a complex multi-chromosomal circular amplification (F).

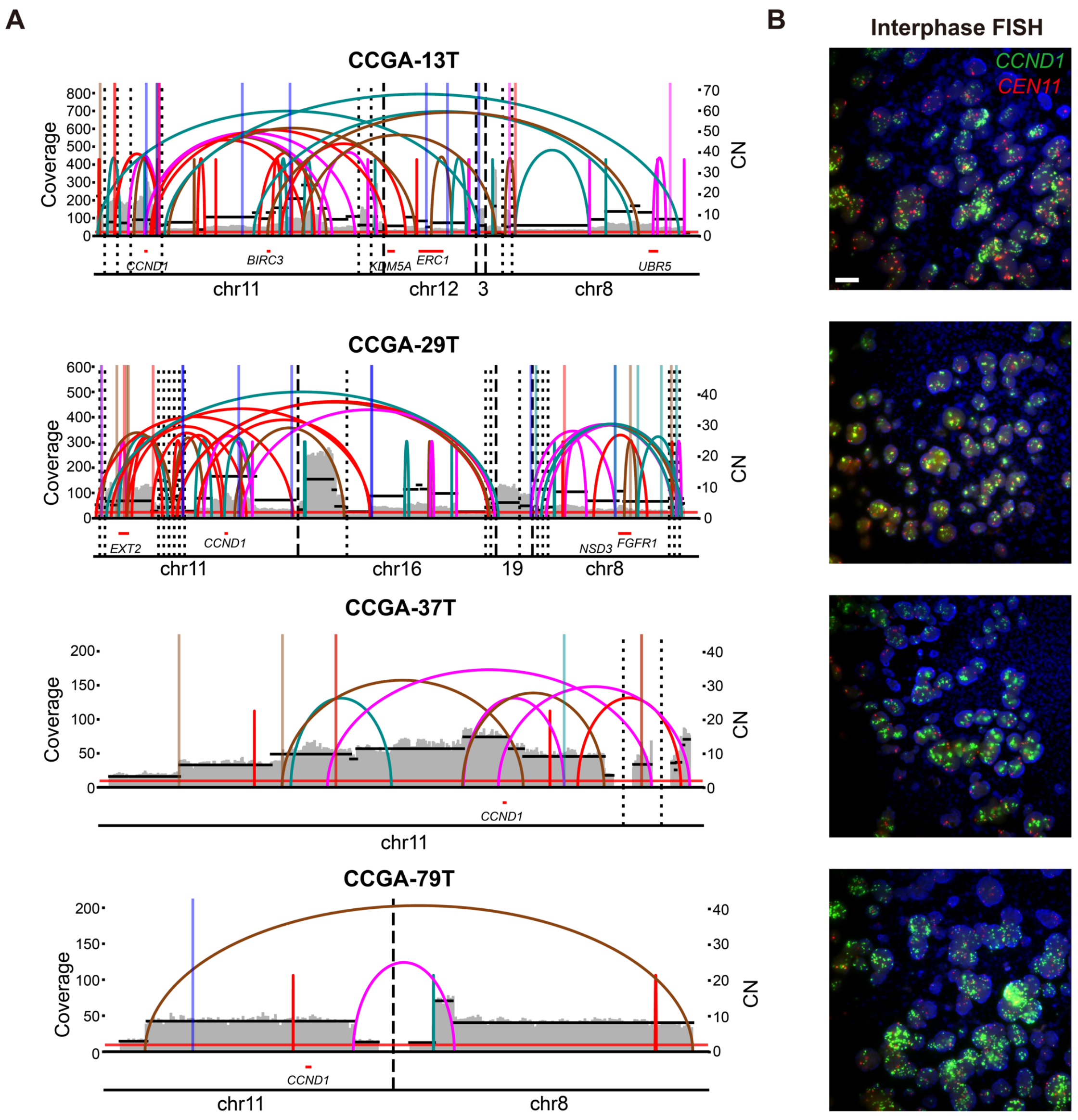

Figure S3. Validation of CCND1 amplification by Interphase FISH

(A) AA-generated structural variant (SV) and breakpoint graph of four *CCND1*-containing ecDNA.

(B) Interphase FISH microscopy targeting the amplified *CCND1* in four available formalin-fixed paraffin-embedded (FFPE) tumor samples. The green and red signals represent the *CCND1* gene and Centromere 11 (*CEN11*) region, respectively. Scale bar, 10 μm.

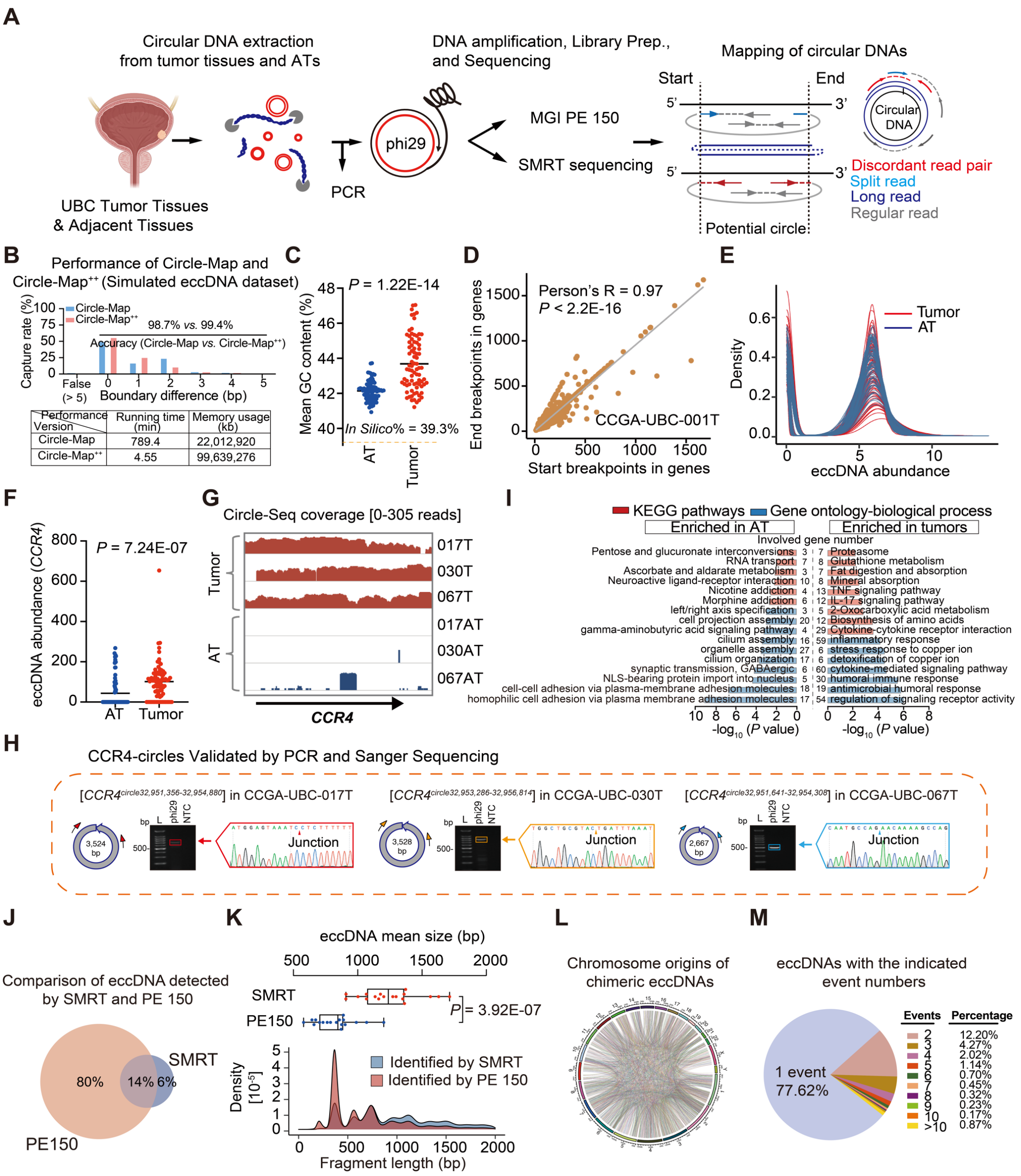

Figure S4. Genome-scale map of small extrachromosomal circular DNAs (eccDNAs) in CCGA-UBC Samples

(A) Circle-Seq method for mapping of eccDNAs. Paired AT-tumor samples were collected from 80 CCGA-UBC patients. Total high molecular weight genomic DNA (HMW *g*DNA) was isolated from tissues using a magnetic-based approach. The linear portions of HMW *g*DNA were hydrolyzed by Exonuclease V (Exo V). PCR was performed to validate the removal of linear DNA. The purified circular DNAs were amplified by phi29 polymerase. The amplified products were subjected to MGI PE150 sequencing (n = 80 pairs) and long-read SMPT sequencing (PacBio) (n = 9 pairs). The eccDNAs were detected from sequencing data by Circle-Map^{++} and the Consensus eccDNA generation method (see methods).

(B) Evaluation of the performance of Circle-Map and Circle-Map^{++} in the detection of eccDNAs. Circle-Map and Circle-Map^{++} were evaluated on a simulated eccDNA dataset. Shown are the capture rate and accuracy of Circle-Map and Circle-Map^{++} (top) and the running time and memory usage of Circle-Map and Circle-Map^{++} (bottom).
(C) Comparison of the mean GC content of the eccDNAs detected in CCGA-UBC tumors and ATs (Paired t-test).
(D) Correlation of the number of start breakpoints and the number of end breakpoints in each gene (CCGA-UBC-001T) (Person correlation test). The start and end breakpoints of each eccDNA refer to the upstream and downstream boundaries of the circularized genomic region.
(E) Density plot showing the eccDNA abundance distribution in ATs (blue) and tumors (red) (n = 80 pairs).
(F) Comparison of the eccDNA abundance of the *CCR4* gene between tumors and ATs (n = 80 pairs; Paired t-test).
(G) Circle-Seq reads coverage across the *CCR4* locus on chromosome 3 in three example cases of paired tumors and ATs.
(H) Validation of three typical *CCR4*-related circles by gel electrophoresis and Sanger sequencing.
(I) Pathways enriched for genes with differential eccDNA abundance between tumors and ATs.
(J) Venn diagram showing the overlap of non-chimeric eccDNAs identified by SMRT and PE150.
(K) Top panel: comparison of the mean length of eccDNA detected by SMRT and PE150 (n = 9 pairs; Paired t-test). Bottom panel: Fragment length distribution of eccDNA detected by SMRT and PE 150 (Polled data from 18 cases from each group).
(L) Circle plot presenting the chromosome origins of all identified chimeric eccDNAs
(M) Pie chart displaying the percentage eccDNA with indicated event numbers. The eccDNAs were detected by SMRT.

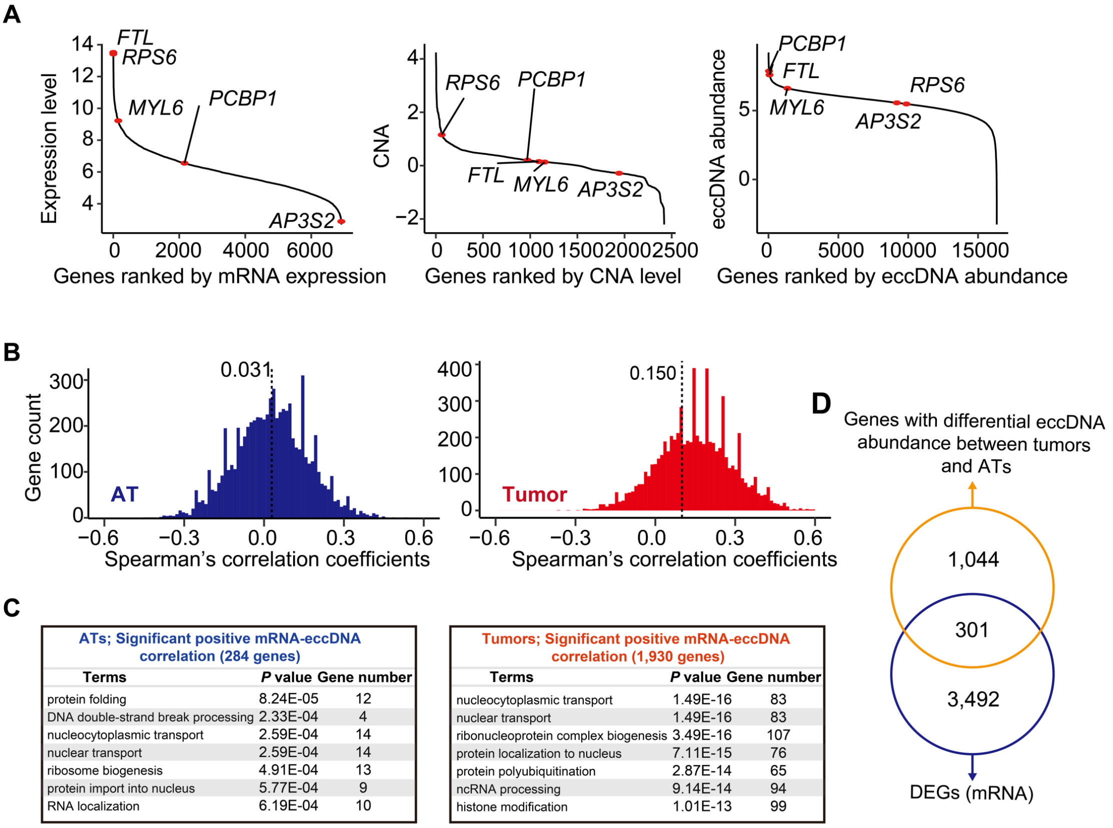

ATs; Significant positive mRNA-eccDNA correlation (284 genes)		
Terms	*P* value	Gene number
protein folding	8.24E-05	12
DNA double-strand break processing	2.33E-04	4
nucleocytoplasmic transport	2.59E-04	14
nuclear transport	2.59E-04	14
ribosome biogenesis	4.91E-04	13
protein import into nucleus	5.77E-04	9
RNA localization	6.19E-04	10

Tumors; Significant positive mRNA-eccDNA correlation (1,930 genes)		
Terms	*P* value	Gene number
nucleocytoplasmic transport	1.49E-16	83
nuclear transport	1.49E-16	83
ribonucleoprotein complex biogenesis	3.49E-16	107
protein localization to nucleus	7.11E-15	76
protein polyubiquitination	2.87E-14	65
ncRNA processing	9.14E-14	94
histone modification	1.01E-13	99

Figure S5. Correlation analysis of eccDNA abundance and mRNA expression at the gene level

(A) Ranked of mRNA expression, eccDNA abundance, and CNA value in CCGA-UBC-065 tumor sample.

(B) Correlation analysis of eccDNA and mRNA (6,918 mRNA-eccDNA pairs) in tumors and adjacent tissues (ATs) using Spearman's correlation.

(C) Pathways enriched in genes with significant eccDNA-mRNA correlation in ATs (left) and tumors (right).

(D) Overlap of genes genes exhibiting significant changes in both gene expression levels and eccDNA abundance in tumors and ATs.

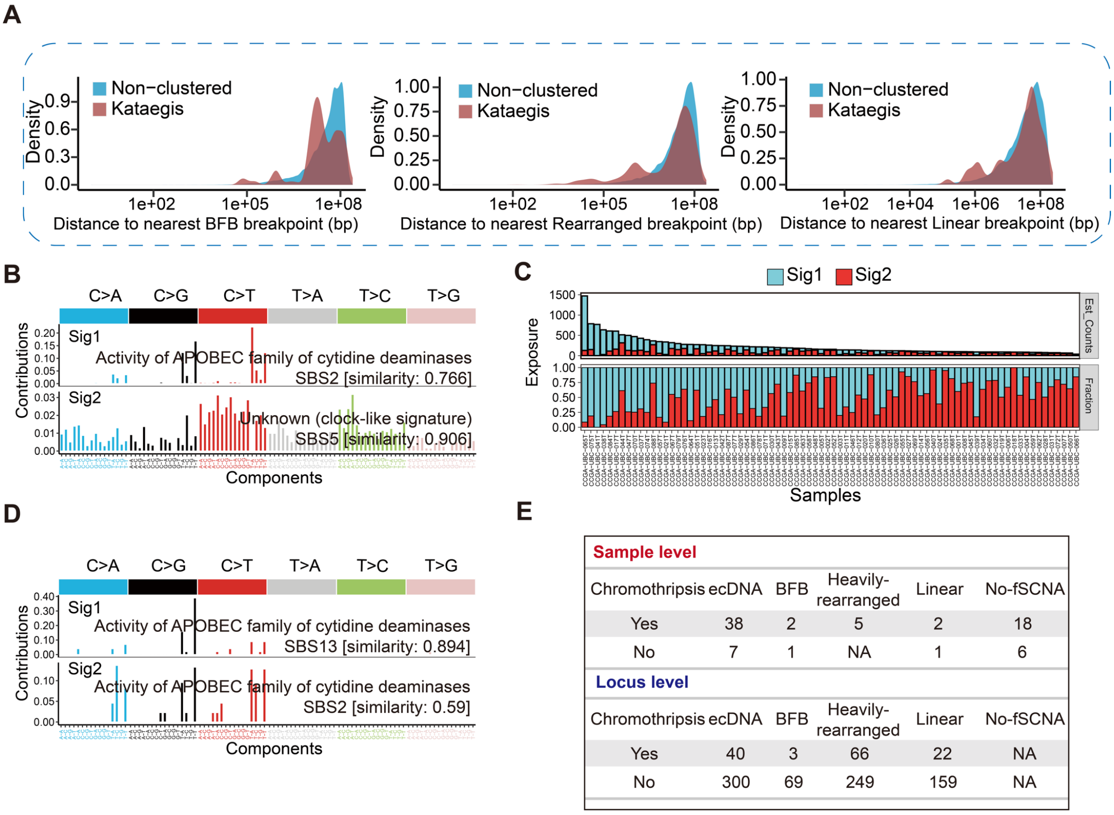

Figure S6. Correlation analysis of ecDNA with clustered somatic mutations and chromothripsis

(A) Distances to the nearest breakpoints for BFB (Left), Heavily-Rearranged (Middle), and Linear (Right) events associated with kataegis mutations versus non-clustered mutations.

(B) Mutational signatures associated with kataegis events.

(C) Number of mutations for each mutational signature (Est_Counts) and their normalized contributions across samples (Fraction).

(D) Mutational signatures of kataegis within 10kb of ecDNA breakpoints

(E) Co-occurrence of chromothripsis with focal amplification. Sample level analysis of chromothripsis distribution across different amplification samples and locus-level overlap between chromothripsis and each type of amplicon,

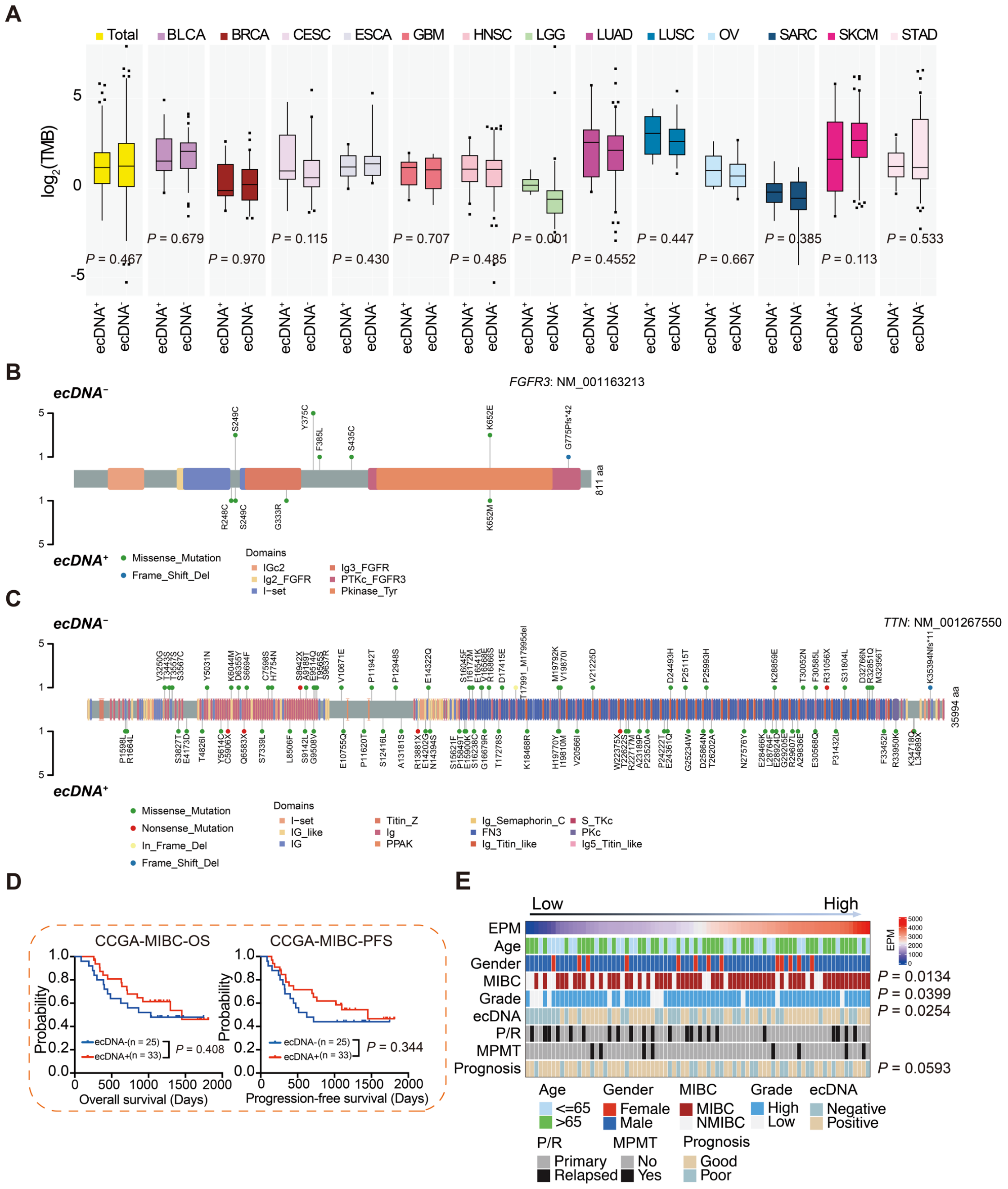

Figure S7. Mutation load in ecDNA[-] and ecDNA[+] tumors and the relationship between ecDNA/eccDNA and clinical variables

(A) Comparison of tumor mutation burden (TMB) between ecDNA[-] and ecDNA[+] tumors across 13 cancer types from TCGA datasets.

(B-C) Lollipop plots showing the locations of somatic mutations in tumors with and without ecDNA for FGFR3 (B) and TTN (C).

(D) Survival analysis of CCGA-MIBC patients stratified by the presence (n = 25) or absence of ecDNA (n = 33) (log-rank test).

(E) Association between eccDNA counts per million mapped reads (EPM) and eight clinical and molecular variables (Wilcoxon rank-sum test).

OS	Variables		n	HR (95%CI)	P value
	Gender	Male	67		
		Female	13	3.3782 (1.4741 - 7.742)	0.00401
	Age	<=65	33		
		>65	47	1.6495 (0.7692 - 3.5374)	0.1985
	MIBC/ NMIBC	NMIBC	22		
		MIBC	58	1.3744 (0.7692 - 3.537)	0.47154
	Grade	Low	13		
		High	67	1.6733 (0.4735 - 5.914)	0.42414
	ecDNA	Negative	35		
		Postive	45	0.9683 (0.4669 - 2.008)	0.93087
	EPM	Low	42		
		High	38	1.7471 (0.8574 - 3.560)	0.12444

PFS	Variables		n	HR (95%CI)	P value
	Gender	Male	67		
		Female	13	2.8762 (1.2535 - 6.599)	0.0127
	Age	<=65	33		
		>65	47	1.375 (0.6497 - 2.910)	0.4051
	MIBC/ NMIBC	NMIBC	22		
		MIBC	58	1.5223 (0.6453 - 3.591)	0.3372
	Grade	Low	13		
		High	67	1.9175 (0.5468 - 6.724)	0.3092
	ecDNA	Negative	35		
		Postive	45	0.9374(0.4580 - 1.907)	0.8528
	EPM	Low	42		
		High	38	1.7337 (0.8706 - 3.452)	0.1174

Figure S8. Multivariate analysis of associations between EPM and clinical variables and prognosis.
